# Molecular targets of Chinese herbs: a clinical study of metastatic colorectal cancer based on network pharmacology

**DOI:** 10.1038/s41598-018-25500-x

**Published:** 2018-05-08

**Authors:** Hongxu Zhu, Jian Hao, Yangyang Niu, Dan Liu, Dan Chen, Xiongzhi Wu

**Affiliations:** 1 Tianjin Medical University Cancer Institute and Hospital, National Clinical Research Center for Cancer, Key Laboratory of Cancer Prevention and Therapy, Tianjin, 300060 China; 2 Tianjin Children’s Hospital, Tianjin, 300134 China; 3 Department of Pharmacology, School of Basic Medical Sciences, Tianjin Medical University, Tianjin, Qi-Xiang-Tai Road, Tianjin, 300070 China; 4 Zhong-Shan-Men Inpatient Department, Tianjin Medical University Cancer Institute and Hospital, Tianjin, 300060 China

## Abstract

Increasing evidence has shown that Chinese herbal medicine (CHM) has promising therapeutic effects in colorectal cancer (CRC); however, the active ingredients and potential targets remain unclear. In this study, we aimed to investigate the relative molecular targets of the Chinese herbs that have been found effective in treating metastatic CRC (mCRC) based on clinical data and network pharmacology. In multivariate analysis CHM resulted an independent prognostic factor. The hazard ratio was 0.103 (95% confidence interval = 0.064–0.164; *P* < 0.001). Compared with the non-CHM group, the median survival time of the CHM group was also improved (40 versus 12 months; P < 0.001). Eighteen out of 295 herbs showed significant correlation with survival results (P < 0.05). Bioinformatics analysis indicated that the 18 herbs realize anti-CRC activity mainly through suppressing the proliferative activity of ERBB2, peroxisome proliferator-activated receptor gamma, and retinoid X receptor, suppressing angiogenesis via inhibition of VEGFR and VEGFA expression, inhibiting the phosphatidylinositol-3-kinase/AKT1 signaling pathway directly through SRC and AKT1, and reducing tumor necrosis factor-induced inflammation.

## Introduction

Colorectal cancer (CRC) is one of the most commonly diagnosed cancers and the third leading cause of cancer-related deaths worldwide^1^. Approximately 20–25% of the patients have distant metastases at the time of diagnosis; in addition, surgery becomes non-beneficial in a large proportion of these patients because of the inconspicuous and nonspecific early symptoms^2–5^. Complete resection (R0) of metastases and primary cancer focus is the main therapy to achieve long-term survival in patients with metastatic CRC (mCRC)^6^. However, patients with unresectable mCRC have a life expectancy of 8 months only^7^. Combination therapy with chemotherapy and targeted therapy could prolong the median survival time of patients with mCRC^8–10^. However, long-term therapy could result in serious adverse reactions and reduce the quality of life. Therefore, the prognosis and quality of life of mCRC patients are still below expectations.

Chinese herbal medicine (CHM), a widely used supplementary and alternative medicine therapies in China, has been shown to reduce the toxic and side effects of radiotherapy and chemotherapy, improve the immune function, reduce postoperative metastasis and recurrence, and relieve tumor-related symptoms^11–13^. In addition, oral CHM could improve the quality of life, prolong the survival rate, enhance the immediate tumor response, and increase the effectiveness of chemotherapy in patients with CRC^14–16^. However, the herbs in the formula that are directly related to survival, as well as their active ingredients and potential targets remain unclear.

Network pharmacology, which clarifies the synergistic effects and underlying mechanisms of multi-component and multi-target agents using the analysis of networks, is a suitable approach to measure the efficacy and to reveal the functional mechanisms of multi-target drugs^17,18^. In recent years, network pharmacology has been developed rapidly, and especially, the concept of “network target” has brought a new era in the field of CHM^19^. It provides a new research paradigm for translating CHM from an experience-based medicine to an evidence-based medicine system, which will accelerate CHM drug discovery, and also improve current drug discovery strategies^20–22^.

**Figure 1:**
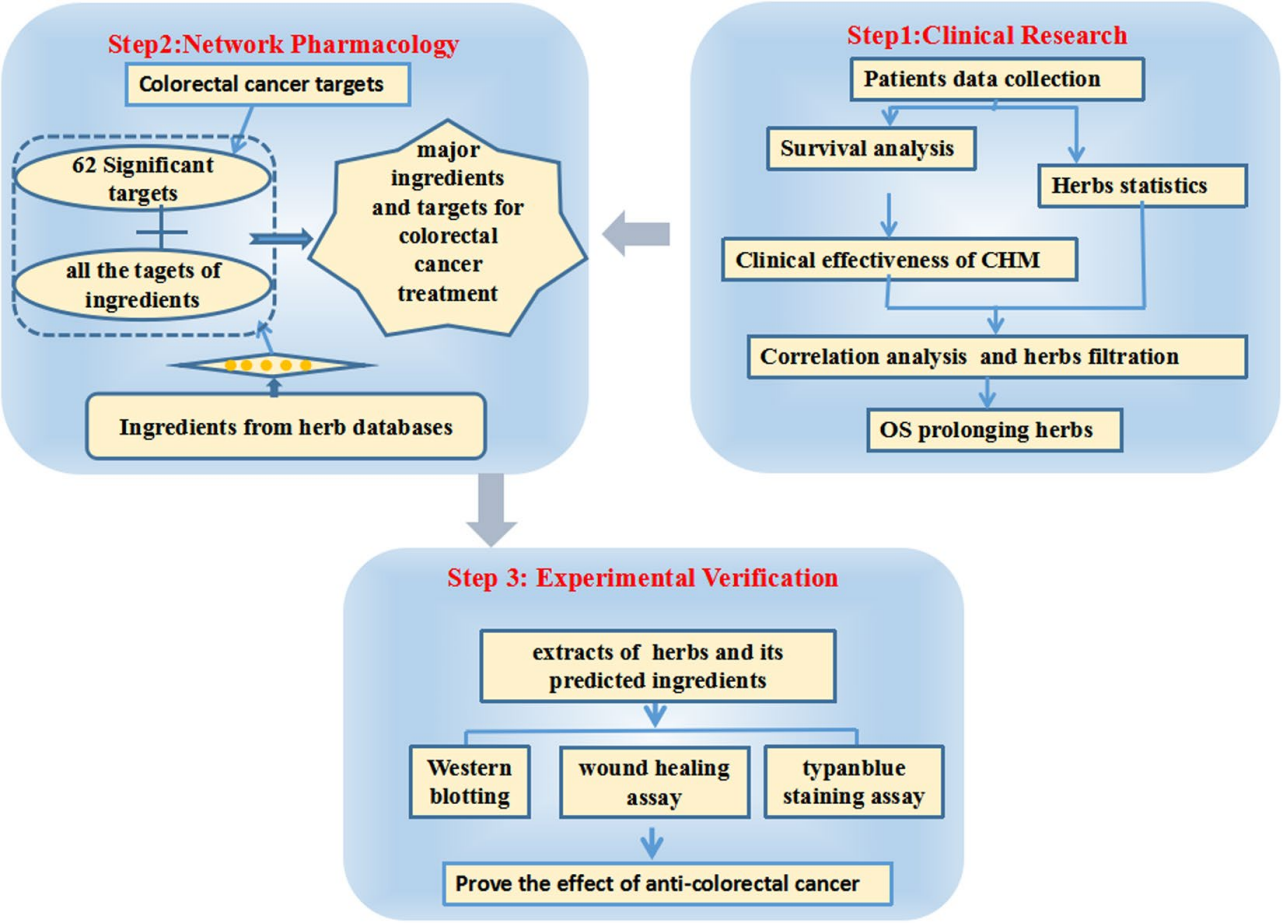
Process overview.

In the present study (Fig. 1), we investigated 222 patients with mCRC to evaluate the efficiency of CHM and identify the effective herbs that were closely correlated with survival. Furthermore, we investigated the underlying pharmacological mechanisms of the effective herbs using bioinformatics approaches.

## Results

### Survival characteristics

In this research, we retrospectively studied 222 patients who were diagnosed with mCRC. Among them, 78 patients received CHM treatment, and 144 patients received non-CHM treatment. All patients exhibited metachronous or simultaneous distant metastases, and complete resection of the cancer was performed in 71 patients. The baselines of the patient demographics were equal between patients with and without CHM treatment (Table S1). Statistical results demonstrated that the patients’ age, gender, smoking (yes/no), family history (yes/no), tumor site (colon/rectum), primary tumor size (≤4/>4 cm), differentiated degree (high/middle/poor), carbohydrate antigen 199 (high/normal), carcinoembryonic antigen (high/normal), lymph node metastases, systemic chemotherapy (yes/no), radiotherapy (yes/no) and R0 after metastasis (yes/no) didn’t differ obviously between the two groups.

Kaplan-Meier analysis indicated that smoking history (*P* = 0.028), primary tumor size (*P* = 0.023), invasion of the serous membrane (*P* < 0.001), primary tumor differentiation (*P* < 0.001), pathological classification (*P* = 0.005), lymph node metastasis (*P* < 0.001), and carcinoembryonic antigen (CEA) >5 ng/mL (*P* = 0.010) were obviously correlated with decreased median survival time. In contrast, systemic chemotherapy (*P* < 0.001), radiotherapy (*P* < 0.001), complete resection after metastasis (*P* < 0.001), and CHM (P < 0.001) were considered to be beneficial factors for median survival time. Cox regression analysis showed that complete resection after metastasis and CHM were independent protective factors. The hazard ratio (HR) was 0.103 (95% CI 0.064∼0.164; P < 0.001). Detailed data are presented in Table 1.

**Table 1:** Univariate and multivariate analyses of variables affecting the survival of 222 patients with mCRC.

Characteristics	Univariate Analysis			Multivariate analysis		
	N (%)	*P* Value	Β	Exp(β)	95% CI for Exp(β)	*P*
**Age(year)**		0.320	—	—	—	—
≤40	14 (6.3)					
40–60	116 (52.3)					
≥60	92 (41.4)					
**Gender**		0.815	—	—	—	—
Male	136 (61.3)					
Female	86 (38.7)					
**Smoking history**		***0.028***	—	—	—	—
Yes	70 (31.5)					
No	152 (68.5)					
**Primary tumor size**		***0.023***	—	—	—	—
≤4	76 (34.2)					
>4 cm	146 (65.8)					
**Primary tumor location**		0.078	—	—	—	—
Rectum	115 (51.8)					
Colon	107 (48.2)					
**Invaded the serous membrane**		***<0.001***	0.794	2.212	1.412–3.464	***0.001***
Yes	169 (76.1)					
No	53 (23.9)					
**Tumor differentiation**		***<0.001***	0.446	1.561	1.108–2.199	***0.011***
High	10 (4.5)					
Middle	171 (77.0)					
Poor	41 (18.5)					
**General classification**		0.653	—	—	—	—
Ulcer type	188 (84.7)					
Uplift type	29 (13.0)					
Infiltrating type	5 (2.3)					
**Pathological classification**		***0.005***	—	—	—	—
Tubular adenocarcinoma	176 (79.3)					
Mucinous adenocarcinoma	34 (15.3)					
Papillary adenocarcinoma	12 (5.4)					
**Lymph node metastases**		***<0.001***	0.134	1.144	0.925–1.415	0.216
No	108 (48.6)					
1–3	79 (35.6)					
≥ 4	35 (15.8)					
**CEA**		***0.010***	0.428	1.534	1.069–2.199	***0.020***
High	129 (58.1)					
Normal	93 (41.9)					
**CA19-9**		***0.001***	—	—	—	—
High	75 (33.8)					
Normal	147 (66.2)					
**CA24-2**		***<0.001***	—	—	—	—
High	105 (47.3)					
Normal	117 (52.7)					
**CA72-4**		***<0.001***	—	—	—	—
High	75 (33.8)					
Normal	147 (66.2)					
**Systemic Chemotherapy**		***<0.001***	−0.823	0.439	0.278–0.695	***<0.001***
Yes	193 (86.9)					
No	29 (13.1)					
**Radiotherapy**		***<0.001***	−1.058	0.347	0.207–0.581	***<0.001***
Yes	45 (20.3)					
No	177 (79.7)					
**Complete resection after metastasis**		***<0.001***	−1.988	0.137	0.086–0.218	***<0.001***
Yes	71 (32.0)					
No	151 (68.0)					
**CHM**		***<0.001***	−2.275	0.103	0.064–0.164	***<0.001***
Yes	78 (35.1)					
No	144 (64.9)					

**CHM**, Chinese herbal medicine; **CEA**, carcinoembryonic antigen; **CA-199**, carbohydrate antigen 199; **CA-242**, carbohydrate antigen 242; **CA-724:** carbohydrate antigen 724.

**Figure 2:**
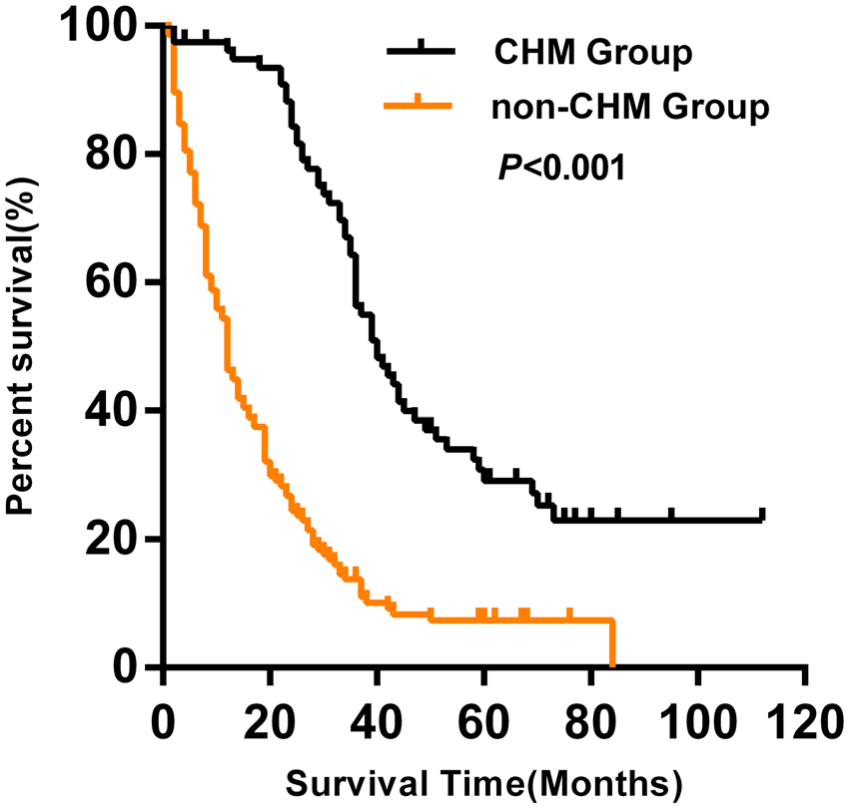
Kaplan–Meier curve between CHM and non-CHM groups. Patients who received CHM treatment had a longer median survival time than those without CHM treatment (40 *vs*. 12 months, *P* < 0.001). **CHM**, Chinese herbal medicine.

Patients in the CHM group had a longer median survival time (40 months) compared with the non-CHM group (12 months) (P < 0.001). In addition, the 1-, 2-, 3-, and 5-year survival rates were 96.1, 84.3, 56.3, and 29.2% in the CHM group *versus* 46.3, 24.5, 13.8, and 7.3% in the non-CHM group, respectively (Fig. 2).

### Candidate targets associated with CRC

**Figure 3:**
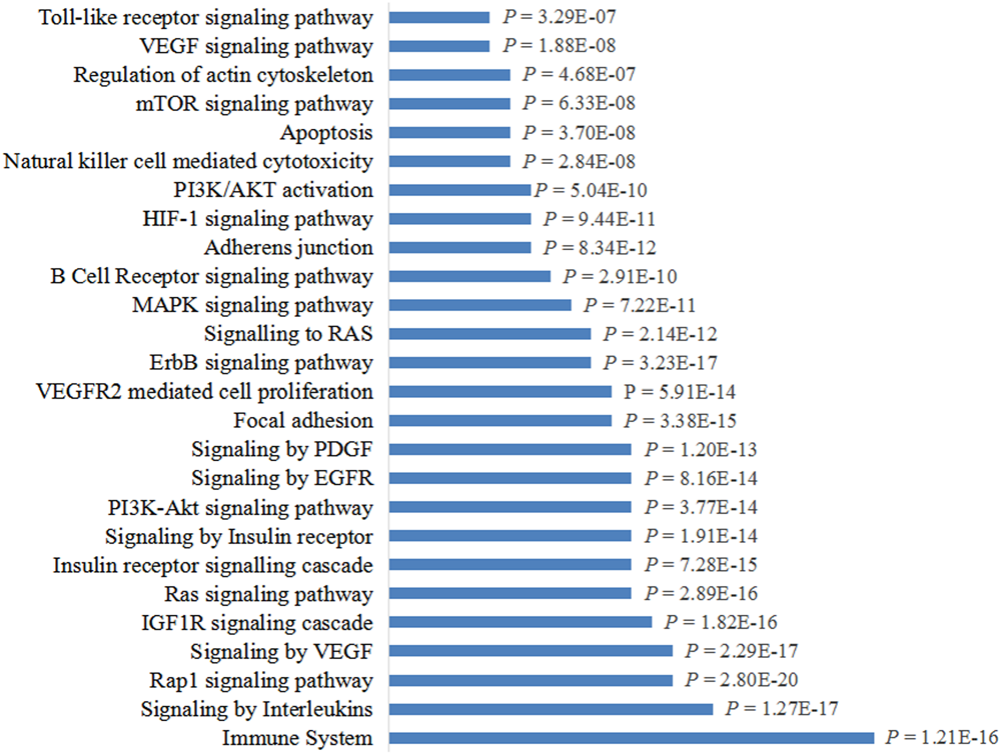
Enrichment analysis of candidate targets for colorectal cancer treatment. Enrichment analysis showed that candidate targets for colorectal cancer treatment were frequently involved in intracellular signaling cascades and immune responses.

Although hundreds of significant genes and proteins have been shown to be differently expressed or to exhibit genetic variations in CRC, only a few of hub genes and proteins were identified as candidate targets. Therapeutic Target Database (TTD) provides information about the known and explored therapeutic protein and nucleic acid targets. Sixty-two significant targets were obtained from the TTD. As shown in Fig. 3, these targets were primarily involved in cell proliferation, cancer metastasis, and immunity. They included the RAS, phosphoinositide 3-kinase (PI3K)/AKT1, vascular endothelial growth factor (VEGF), and interleukin signaling pathways, as well as pathways involved in focal adhesion.

### Candidate herbs related to CRC and their putative major ingredients and targets

**Figure 4:**
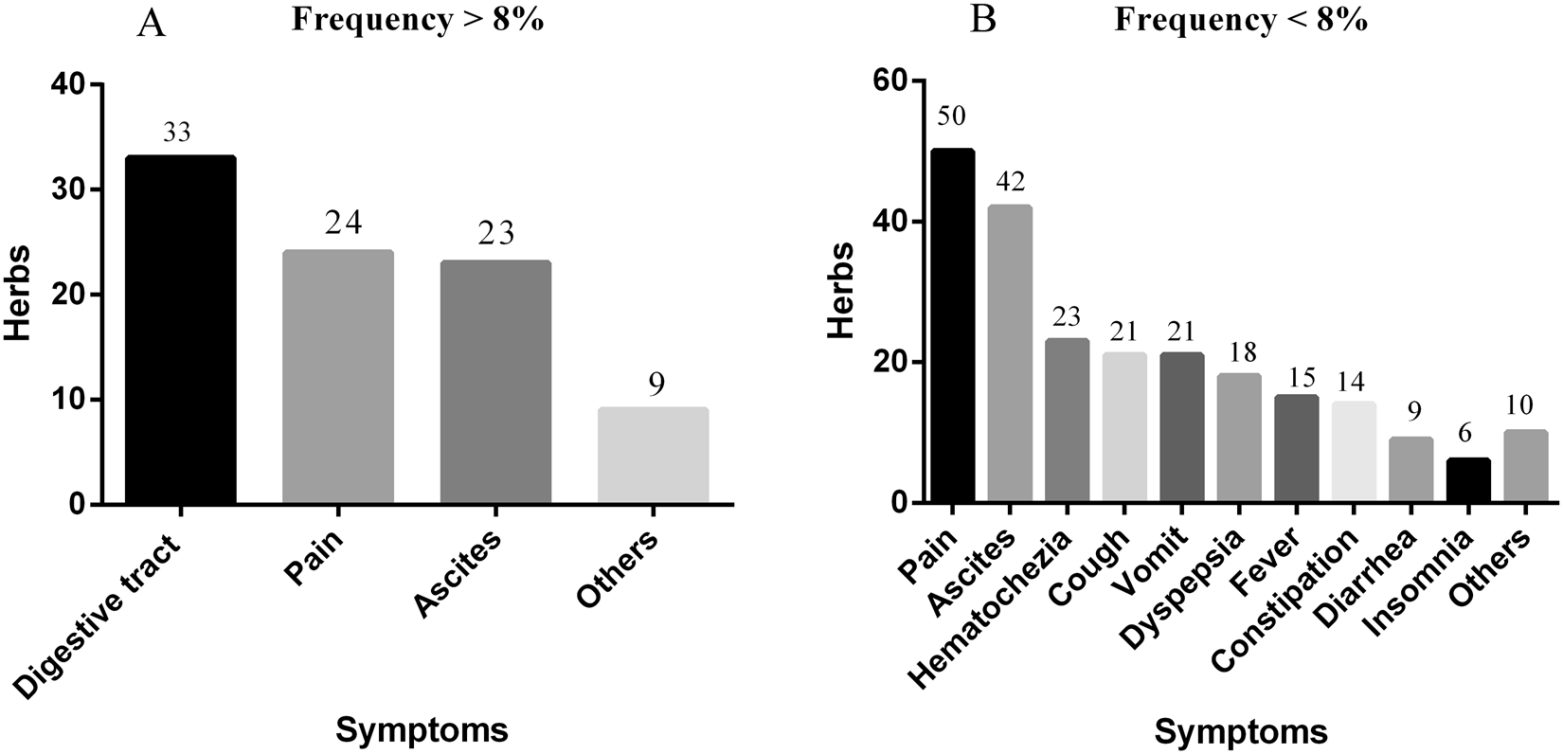
Analysis of the herbs unrelated to survival. (**A**) Seventy-four commonly used herbs unrelated to survival were applied to relieve the major complications of CRC mainly through relieving pain, diuresis and alleviating digestive tract symptoms. (**B**) 203 herbs uncommonly used herbs were administered to relieve various uncomfortable symptoms.

A total of 78 patients received CHM treatment, and all the CHM prescriptions used by them were counted; these prescriptions contained 295 herbs. Among the 295 types of herbs, we distinguished the commonly used herbs from the uncommonly used herbs according to the single herb frequency/total frequency. A total of 92 herbs with using frequency/total frequency >8% were defined as commonly used herbs, and 18 herbs of these were closely associated with survival. Other 74 commonly used herbs were applied to relieve the major complications of CRC mainly through relieving pain, diuresis and alleviating digestive tract symptoms (Fig. 4A). Furthermore, a total of 203 herbs with using frequency <8% were defined as uncommonly used herbs, which were frequently administered to relieve various uncomfortable symptoms, such as pain, ascites, vomit, hematochezia, cough, expectoration, fever, dyspepsia, constipation, diarrhea, insomnia and so on (Fig. 4B). The maximum value of single herb frequency was 1570. And 92 herbs with using frequency >125.6 (1570 × 8%) were selected for correlation analysis. Statistical results indicated that 18 herbs were closely related to improving survival(*P* < 0.05, correlation coefficients ≧ 0.23). These 18 herbs were *Lycii Fructus* (LF), *Magnolia officinalis Rehd Et Wils* (MO), *Radix Clematidis* (RC), *Aucklandiae Radix* (AR), *Angelicae sinensis Radix* (ASR), *Xanthii Fructus* (XF), *Eriocauli Flos* (EF), *Cassiae Semen* (CaS), *Fallopia multiflora* (FM), *Selaginella doederleinii Hieron* (SDH), *Herba Patriniae* (HP), *Portulacae Herba* (PH), *Coicis Semen* (CoS), *Taraxacum mongolicum Hand* (TM), *Agrimonia eupatoria* (AE), *Ranunculi ternati Radix* (RTR), *Schisandrae chinensis Fructus* (SCF), *Radix Paeoniae rubra* (RPR). Furthermore, 165 ingredients present in these 18 herbs were suggested to be related to CRC treatment. To further elucidate the underlying molecular mechanisms of these herbal medicines, targets of the proposed active ingredients were identified based on a comprehensive method. These candidate ingredients yielded 41 potential targets involved in CRC.

### Targets prediction in the candidate ingredient-target network

**Figure 5:**
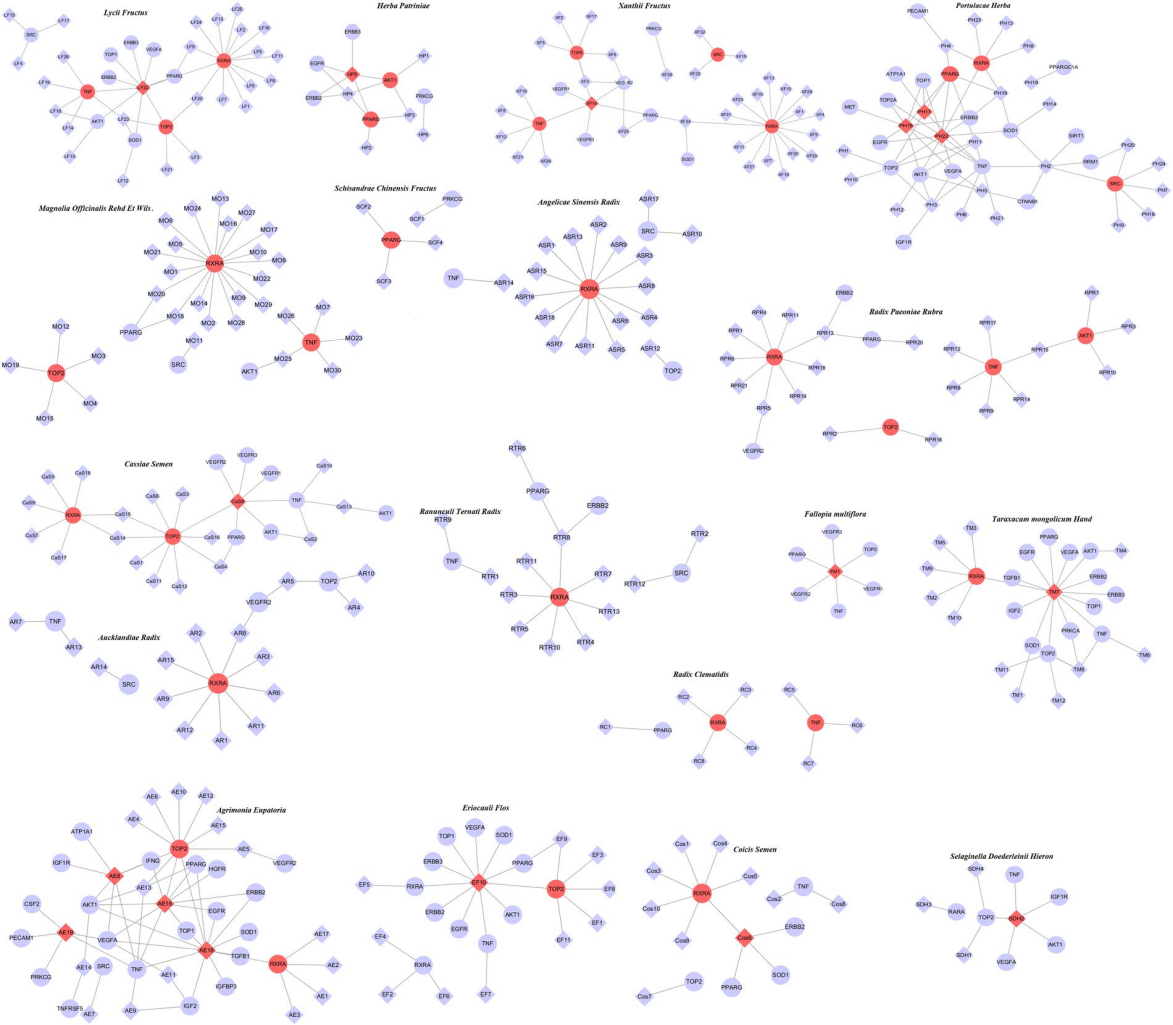
The ingredient-target networks. The diamond nodes represent ingredients, the circular nodes represent targets, and the nodes with red color are the major ingredients and targets involved in CRC treatment. **LF**, *Lycii Fructus*; **MO**, *Magnolia officinalis Rehd Et Wils;* **RC**, *Radix Clematidis*; **AR**, *Aucklandiae Radix*; **ASR**, *Angelicae sinensis Radix*; **XF**, *Xanthii Fructus*; **EF**, *Eriocauli Flos*; **CaS**, *Cassiae Semen*; **FM**, *Fallopia multiflora*; **SDH**, *Selaginella doederleinii Hieron*; **HP**, *Herba Patriniae*; **PH**, *Portulacae Herba*; **CoS**, *Coicis Semen*; **TM**, *Taraxacum mongolicum Hand*; **AE**, *Agrimonia eupatoria*; **RTR**, *Ranunculi ternati Radix*; **SCF**, *Schisandrae chinensis Fructus*; **RPR**, *Radix Paeoniae rubra*.

**Figure 6:**
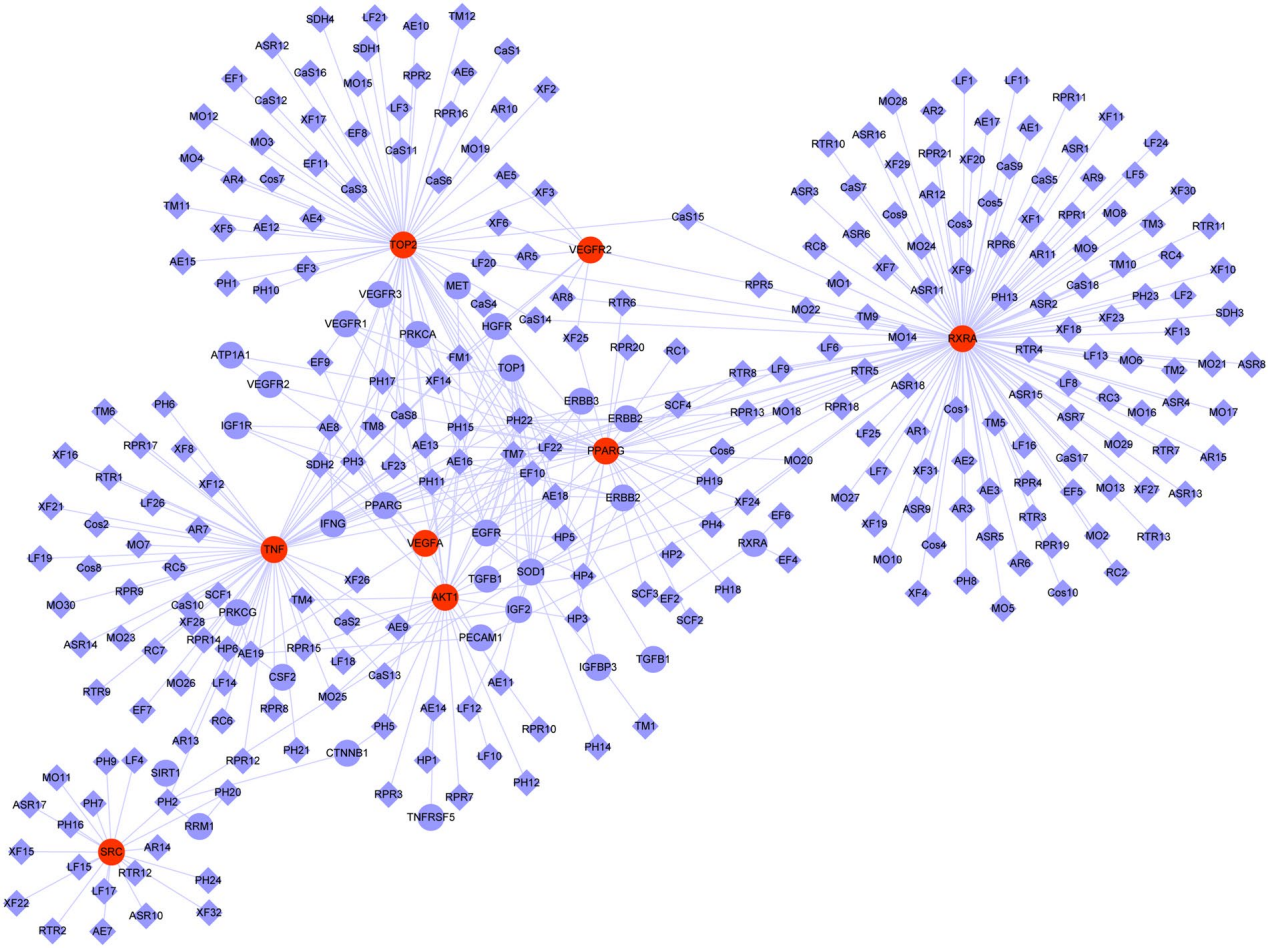
Network of all the candidate ingredients and candidate protein targets of the 18 herbs for CRC treatment. The diamond nodes represent ingredients, the circular nodes represent targets, and the nodes with red color are the major ingredients and targets involved in CRC treatment.

The 18 herbs contain 309 compounds, 165 of which have certain effect on CRC. Among them, 18 ingredients corresponded to the most targets and exhibited high scores, and each of them hit the 41 major putative colorectal cancer targets. Ingredient-target networks of the herbs are shown in Fig. 5. Targets in the outer circle had much fewer interactions with the candidate ingredients than those in the inner circle, which also indicated that many candidate targets were affected by only one candidate ingredient. Alternatively, some targets could be modulated by multiple rather than a single ingredient. We constructed a general network of all the candidate ingredients and candidate protein targets of the 18 herbs. As shown in Fig. 6, the major ingredients and targets involved in CRC treatment include SRC, AKT1, VEGFA, VEGFR, TNF, TOP2, PPARG, RXRA, which are represented by the nodes with red color. The putative major ingredients were determined by analyzing the topological parameters of the networks (Table 2). Quercetin might play an important role in CRC treatment since it is the major ingredient of five herbs (*Lycii Fructus, Eriocauli Flos, Portulacae Herba, Taraxacum mongolicum Hand, and Phytolaccae Radix*). Ingredients such as emodin, stigmasterol, apigenin, and oleic acid may also play significant roles, because these ingredients were present in more than two herbs. The number of ingredients, serial number of each ingredient, DL values, and number of validated/predicted targets are shown in Table S2.

**Table 2:** The major ingredients and major targets of the 18 herbs involved in CRC treatment.

Chinese Name	Latin name	Major ingredients	Number of targets	Major targets	Frequency (%)	Average dosage(g)	Correlation coefficient	P value
	*Lycii Fructus*	Quercetin (LF22)	11	TNF RXRA TOP2	12.6	10	0.35	0.002
	*Magnolia Officinalis Rehd Et Wils*.	Honokiol (MO18) Magnolol (MO20)	6	TNF RXRA TOP2	15.2	6	0.346	0.003
	*Radix Clematidis*	Nonane (RC6) Palmitic acid (RC7) Stigmasterol (RC8)	3	TNF RXRA PPARG	24.2	10	0.315	0.007
	*Aucklandiae Radix*	(R)-linalool (AR5)	5	RXRA	43.5	5	0.282	0.017
	*Angelicae Sinensis Radix*	Stigmasterol (ASR16)	4	RXRA	52.0	10	0.28	0.018
	*Xanthii Fructus*	Emodin (XF14)	10	TNF RXRA TOP2 SRC	11.5	9	0.274	0.021
	*Eriocauli Flos*	Quercetin (EF10)	12	TOP2	18.5	30	0.274	0.021
	*Cassiae Semen*	Emodin (XF8)	9	RXRA TOP2 TNF	16.6	10	0.274	0.021
	*Fallopia multiflora*	Emodin (FM1)	6	TOP2 TNF	10.2	30	0.274	0.021
	*Selaginella Doederleinii Hieron*	Apigenin (SDH2)	6	TOP2	31.1	30	0.272	0.022
	*Herba Patriniae*	luteolin (EF4)	6	AKT1 PPARG	71.0	30	0.271	0.022
	*Portulacae Herba*	Luteolin (PH15) Myricetin (PH17) Quercetin (PH22)	20	RXRA PPARG SRC	33.6	30	0.263	0.027
	*Coicis* *Semen*	Oleic acid (CoS6)	6	RXRA	14.7	30	0.254	0.032
	*Taraxacum mongolicum Hand*	Quercetin (TM7)	14	RXRA TOP2	15.3	10	0.247	0.038
	*Phytolaccae Radix*	Quercetin (PR8) Apigenin (PR16) Luteolin (PR18) Ursolic acid (PR19)	23	RXRA TOP2	71.8	30	0.246	0.039
	*Ranunculi Ternati Radix*	Oleic acid (RTR8)	5	RXRA	60.4	30	0.244	0.04
	*Schisandrae Chinensis Fructus*	Protocatechuic acid (SCF1) Arnebin 7 (SCF2) Gomisin T (SCF3) Schisanhenol (SCF4)	2	PPARG	42.0	6	0.244	0.041
	*Radix Paeoniae Rubra*	Oleic acid (RPR13) Paeonol (RPR15)	7	RXRA TOP2 TNF AKT1	8.9	20	0.236	0.048

### Holistic mechanisms of anti-CRC medicinal herbs

**Figure 7:**
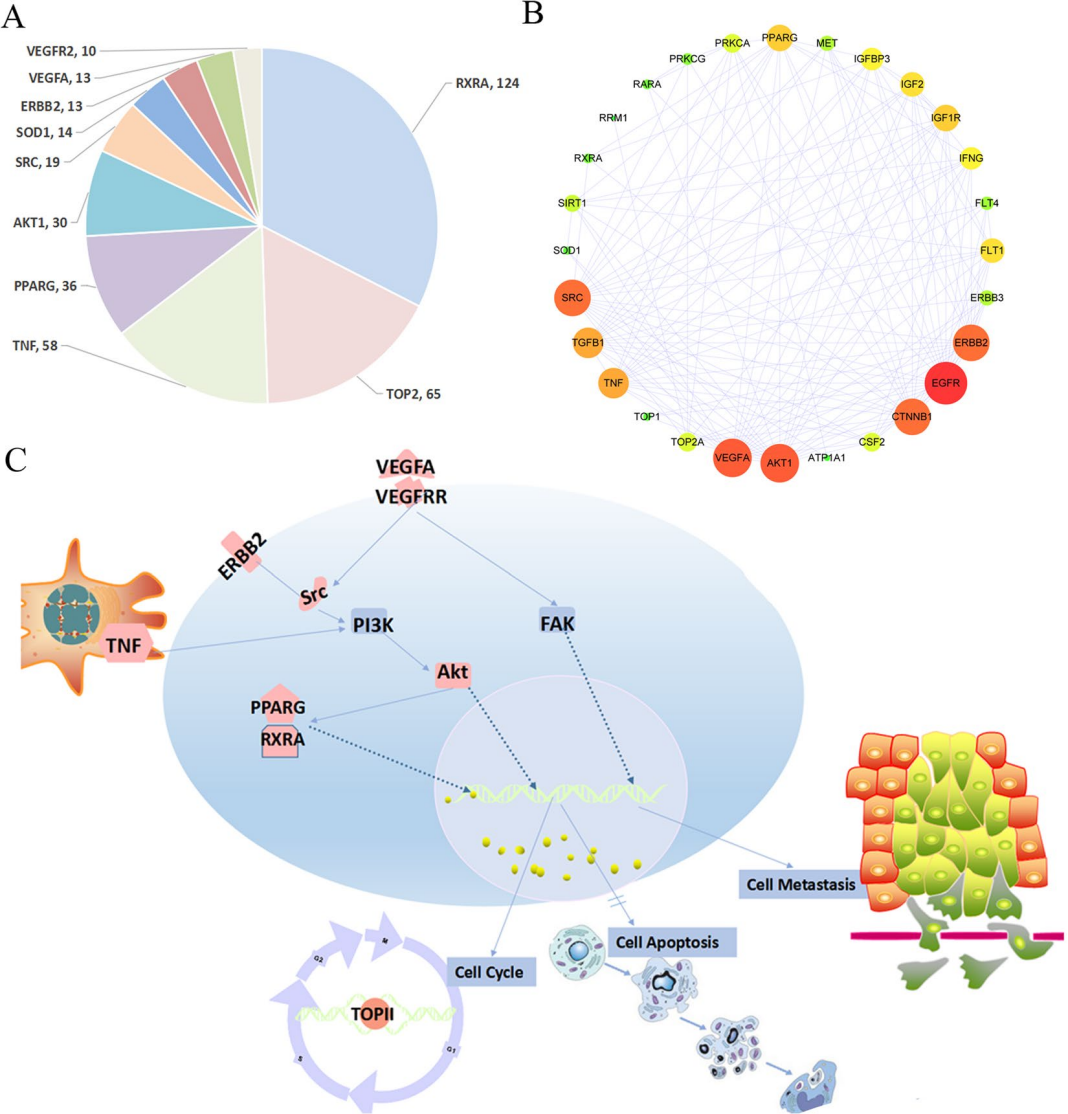
Signaling pathways involved in the actions of the 18 herbs against colorectal cancer. (**A**) Major predicted targets (could be targeted by more than 10 ingredients) were listed according to the scores from high to low. (**B**) Protein-protein interaction of all the candidate protein targets. The map node size gets larger with increased degree and the map color gets bright from green to red with increased degree. This figure was drew using cytoscape with the protein-protein interactions getting from STRING^48,49^. (**C**) The 18 herbs might exhibit anti-colorectal cancer activity mainly *via* 1) inhibition of the proliferative activity of ErbB2, PPARγ, and RXR, 2) suppression of angiogenesis by inhibiting VEGFR and VEGFA expression, 3) inhibition of the PI3K/Akt signaling pathway directly through Src and Akt, and 4) reduction of TNF-induced inflammation.

Cancer is a complicated disease, wherein many parallel signaling pathways are involved in the development and maintenance of tumors. In this research, 41 tumor-associated proteins involved in tumorigenesis were identified as targets of 18 herbs using network analysis. Interestingly, RXRA, TOP2, TNF, PPARγ, AKT1, SRC, ERBB2, VEGFR, and VEGFA had the most of the direct interactions with these herbs, suggesting that these proteins might play important roles in the treatment of CRC. Based on the network and multi-target computational approach, we found that simultaneous manipulation of multiple targets involved in proliferation, such as epidermal growth factor receptor (EGFR), hepatocyte growth factor receptor (HGFR), peroxisome proliferator-activated receptors (PPARs), ERBB2, and insulin-like growth factor 1 receptor (IGF1R), as well as angiogenesis, such as VEGF receptor (VEGFR), might underlie the beneficial effects of the 18 herbs in CRC. Retinoid X receptor A (RXRA), topoisomerase 2 (TOP2), tumor necrosis factor (TNF), PPARγ, AKT1, SRC, ERBB2, VEGFR, and VEGFA were the most important targets because they were inhibited by multiple components of 18 herbs. The major targets and the number of the related ingredients were shown in Fig. 7A. The protein-protein interaction of all the candidate protein targets is shown in Fig. 7B. It indicated that VEGFA, AKT1, EGFR, ERBB2 and SRC played central roles in the protein interactions. Interestingly, these proteins also the major targets of the 18 herbs and play important roles in CRC treatment. To better understand the target functions associated with the 18 herbs, we mapped the targets to the canonical signaling pathways identified in the Kyoto Encyclopedia of Genes and Genomes (KEGG) and summarized the most relevant pathways (Fig. 7C).

### Experimental validation

**Figure 8:**
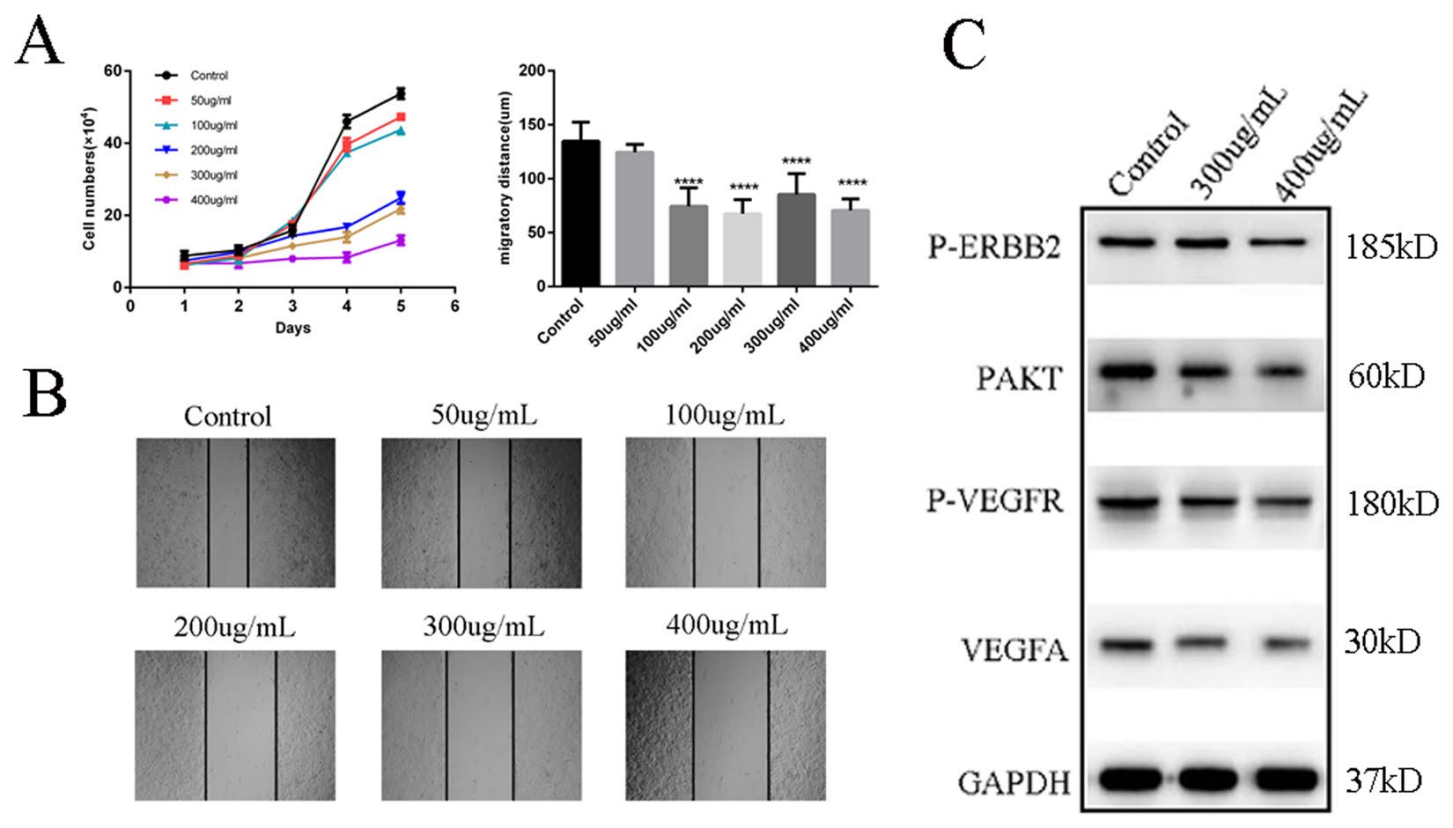
Effect of aqueous extract of 18 herbs on cell proliferation, cell migration and predicted targets. (**A**)The statistical views of cell proliferation (left) and cell migration (right). P-values are represented as asterisks (****P < 0,001). (**B**) The result of wound healing assay. (**C**) Western blot assay analyzed P-ERBB2, P-AKT1, VEGFA and P-VEGFR after the treatment of aqueous extract of 18 herbs. No grouping of gels/blots cropped from diferent parts of the same gel, or from diferent gels, felds, or exposures was performed.

The survival closely associated 18 herbs and their putative targets were validated by experiments. The anti-proliferation cancer effects of 18 herbs were evaluated using typan blue staining assay. The anti-migration effects were evaluated by wound healing assay. The activity of the core predicted targets were tested using western blotting. Figure 8A shows a statistical chart of anti-proliferation and anti-migration of 18 herbs; and the effects of herbs on wound healing assay were shown in Fig. 8B. The experimental results indicated that aqueous extracts of 18 herbs showed a significant suppression effect on cell proliferation after 36 hours in dose of 200 ug/mL, 300 ug/mL and 400 ug/mL *in vitro*. And the aqueous extract could also significantly suppress cell migration at 12 hours in dose of 100 ug/mL, 200 ug/mL, 300 ug/mL and 400 ug/mL. Interestingly, we also found that aqueous extracts of 18 herbs could obviously inhibit cell migration at the 12th hour, but it can only obviously inhibit cell proliferation after 36 hours. It is indicated that aqueous extracts of 18 herbs could affect cell migration in a short time, but it takes enough time to play the role of anti-cell proliferation. Furthermore, aqueous extracts of 18 herbs affected the expression of VEGFA as well as the phosphorylation progress of ERBB2, AKT and VEGFR. As shown in Fig. 8C, aqueous extracts of 18 herbs obviously decreased VEGFA, p-ERBB2, p-AKT and p-VEGFR at the dose of 300 ug/mL and 400 ug/mL.

## Discussion

Previous studies have shown that chemotherapy is usually combined with CHM to improve the resistance of patients, adjust their nutritional imbalance, play an anticancer effect, and facilitate the implementation of the chemotherapy process^23,24^. In addition, previous clinical studies have suggested that CHM treatment in CRC has promising effects. In line with this, the present study showed encouraging results of CHM treatment for colorectal cancer patients. Patients in the CHM group had a longer median survival time (40 months) than those in the non-CHM group (12 months). Compared with non-CHM group, the overall survival rate of CHM group improved significantly. The effects of CHM in mCRC have been previously reported; however, the molecular mechanisms remain to be clarified.

From the TTD, we determined 62 targets related to CRC. And these targets are frequently involved in intracellular signaling cascades that are related to cancer proliferation and metastasis. These pathways included RAP, IGF1R, RAS, and PI3K/AKT1 signaling pathways. These candidate targets are also involved in immune responses, such as natural killer cell-mediated cytotoxicity, B cell receptor signaling pathway, and toll-like receptor signaling pathway.

Clinically, 18 herbs obviously improved the survival of patients with mCRC. 309 complex components were contained in these 18 herbs, and 165 components with potential effects on CRC were preserved for further study. Among the 165 chemical ingredients, 18 ingredients corresponded to the most targets and exhibited high scores. These ingredients were linalool, apigenin, arnebin, emodin, gomisin T, honokiol, luteolin, magnolol, myricetin, nonane, oleic acid, paeonol, palmitic acid, protocatechuic acid, quercetin, schisanhenol, stigmasterol, and ursolic acid. Some of these ingredients, such as quercetin^25,26^, ursolic acid^27,28^, and stigmasterol^29^, were previously shown to exert favorable anticancer activities.

Among the 41 major putative targets of the 18 herbs, RXRA, TOP2, TNF, PPARγ, AKT1, SRC, ERBB2, VEGFR, and VEGFA had the most of the direct interactions with these herbs, suggesting that these proteins might play important roles in the treatment of CRC. RXR and PPARγ are potential candidate targets for cancer prevention and treatment. Once activated, PPARγ binds to RXR to form PPAR–RXR heterodimer. The activation of PPARγ results in growth arrest of colon carcinoma cells *via* induction of cell-cycle arrest or/and apoptosis^30–32^. TOP2 is involved in critical processes in the cell, including DNA replication, transcription, and chromosome segregation. Interfering with TOP2 and generating enzyme-mediated DNA damage are effective strategies for cancer therapy^33^. Chronic inflammatory diseases are associated with an increased risk of CRC^34^. TNF is crucial for the initiation and progression of colitis-associated colon carcinogenesis^35^. TNF antagonists were shown to inhibit inflammatory cytokines, matrix metalloproteinases (MMPs), angiogenesis, and leucocyte trafficking to the sites of inflammation. All these effects could be useful in the treatment of cancer^36^. Activation of AKT1 signaling and inhibition of the expression of phosphatase and tensin homolog (PTEN), a negative regulator of AKT1, have been reported in 60–70% of patients with CRC^37^. Inhibitors of PI3K/AKT1 signaling pathway have been suggested as potential therapeutic agents in CRC^38^. In addition, SRC is elevated in the premalignant tissues in CRC, which could result in induction of apparent loosening of the clusters of colon cancer cells^39^. Overexpression of cytoplasmic ERBB2 plays an important role in the progression of CRC, where its expression correlates with the tumor size, subserosal invasion, liver metastasis, and Dukes’ classification^40^. Moreover, these targets were mapped to the canonical signaling pathways identified in the KEGG. Collectively, our results showed that 18 herbs in CHM might exhibit anti-CRC activity mainly *via* 1) inhibition of the proliferative activity of ERBB2, PPARγ, and RXR; 2) suppression of angiogenesis by inhibiting VEGFR and VEGFA expression; 3) inhibition of the PI3K/AKT1 signaling pathway directly through SRC and AKT1; and 4) Reduction of TNF-induced inflammation.

The anti-colorectal cancer effects of 18 herbs were evaluated by wound healing assay and typanblue staining assay. The experimental results showed that aqueous of 18 herbs had obvious inhibitory effects on cell proliferation and the effects were improved with increased dosages. Cell migration was significantly inhibited (in 12 hours) when the dose of aqueous reached 100 ug/mL *in vitro*. Furthermore, the results of western blotting confirmed the effects of these herbs on predicting targets.

Network pharmacology is a suitable approach to measure the efficacy and to reveal the functional mechanisms of multi-target drugs. At the same time, we must recognize its shortcomings. (i) The components of Chinese medicine screened out by DL value may be incompatible with the exact components; (ii) Because the accuracy of targets prediction tools is different, the results obtained by different prediction tools may be incompatible. Moreover, the number of statistical clinical cases in the present study is not large enough; and the Spearman’s bivariate correlate analysis was adopted to obtain the strong correlated herbs for positive effectiveness, which may not accurately reflect the real clinical situation.

## Conclusion

In the present study, we showed that CHM treatment could significantly improve the survival of patients with mCRC; and correlation analysis identified 18 herbs with positive effects on survival. Moreover, we performed a network pharmacological approach to investigate the underlying mechanisms, which provides a helpful method for herbal research based on clinical data.

## Materials and Methods

### Patient characteristics

The present research was approved by the Ethics Committee of Tianjin Medical University(The certificate no. Tmuhmec2015007). All methods were in accordance with the relevant guidelines and regulations. The ethics committee approved the exemption from informed consent, because this is a retrospective study and most of the patients died before conducting the research. The patients were included to our research by the following criteria: age ≥ 18 years old, clear pathological diagnosis of surgery or colonoscopy, and Chinese herbal medicine treatment in CHM group ≥ 2 months. And the cases were excluded through the following criteria: serious disease, concurrent cancer, incomplete medical records, lack of accurate documentation of the recurrence time, no distant metastasis, and loss to follow-up.

Finally, the medical records of 222 patients diagnosed with mCRC between November 2007 and April 2012 were retrospectively reviewed. Seventy-eight patients who received CHM ≥ two months were assigned to the CHM group, and 144 patients who did not receive CHM were included in the non-CHM group.

### Treatment

CHM group patients received both traditional Chinese medicine (TCM) and Western medicine (WM), and non-CHM group patients received WM only. Radical resection was offered to patients with resectable hepatic metastases. In the CHM group, CHM was administered according to the syndrome differentiation, wherein the formula was administered orally three times daily 30 minutes after meals for 2 months or longer.

Generally, each formula for mCRC included 20–30 kinds of herbs. We counted the prescriptions of CHM from 78 patients, who received TCM treatment with CHM for 24850 days, including 295 herbs. Among the 295 types of herbs, some herbs were frequently used, while some herbs were not commonly used. The commonly used herbs were shared among most patients and were closely associated with survival. The uncommonly used herbs were frequently administered to relieve various uncomfortable symptoms, such as pain, ascites, vomit, hematochezia, cough, expectoration and so on. We adopted the using frequency to identify commonly used herbs. The herbs with frequency >8% were selected for bivariate correlation analysis. In addition, we calculated the coefficients of correlation between each separate herb and survival time. These herbs were used in a further network pharmacology dissection according to the following criteria: single medicinal substance frequency/total frequency >8% and *P* value < 0.05 based on the results of the correlation analysis.

### Significant targets of CRC

Candidate targets related to CRC were obtained from the Therapeutic Target Database (http://bidd.nus.edu.sg/group/cjttd/ttd_home.asp, Version 4.3.02 release on Sep 15th, 2013)^41^.

### Herb formulation ingredient collection, target fishing, and function scoring

The chemical ingredients of herbs and their predicted targets were obtained from the TCM Systems Pharmacology (TCMSP) Database (http://lsp.nwu.edu.cn/tcmsp.php)^42^, then they were selected for further research. DL is a qualitative concept used in drug design for an estimate on how ‘drug-like’ a prospective compound is. The ‘drug-like’ level of the compounds is 0.18, which is used as a selection criterion for the ‘drug-like’ compounds in the traditional Chinese herbs^43,44^. Therefore, the chemical ingredients with DL values ˃ 0.18 were selected for further research.

### Network construction and analysis

The methods applied for network construction and analysis were similar to those used in our previous studies^45–47^. Briefly, the ingredient-target networks of herbs and herb-target networks were constructed using Cytoscape software^48^ (Version 3.2.2) and were analyzed by using Cytoscape plugin CentiScaPe^49^. We finally predicted the main components and targets through calculating the optimal topological structure and analyzing statistical properties of network.

### Experimental validation

Colorectal carcinoma cell line HT29 was used. The anti-colorectal cancer effects of aqueous extract of 18 herbs were tested. In this study, we extracted the aqueous extracts of 18 herbs together. Cell proliferation and cell migration were evaluated using typanblue staining assay and wound healing assay, respectively. The following antibodies were used: p-ERBB2 (Immuno Way, USA), GAPDH (Immuno Way, USA), p-AKT1 (Immuno Way, USA), p-VEGFR (Immuno Way, USA), VEGFA (Abcam, USA) to prove the predicted targets with western blotting. No grouping of gels/blots cropped from diferent parts of the same gel, or from diferent gels, felds, or exposures was performed.

### Statistical analysis

The overall survival (OS) was defined as the time from the diagnosis of mCRC to the day of death or the last follow-up of patients with CRC. Baseline characteristics were compared by the chi-square test. Kaplan Meier method was used for survival rate. Prognostic factors were predicted by multivariate Cox regression analyses. The herbs related to survival were determined by Spearman’s bivariate correlation analysis. P < 0.05 was considered statistically significant. Statistical analyses were carried out by SPSS 21.0.

### Ethical statement

All experimental protocols were under the approval of the Ethics Committee of Tianjin Medical University. (Study number: Tmuhmec2015007).

## Electronic supplementary material

Table S1

Table S2
