# Supplementary material for: Molecular targets of Chinese herbs: a clinical study of metastatic colorectal cancer based on network pharmacology
Source: Sci Rep. 2018 May 8;8:7238. doi: 10.1038/s41598-018-25500-x (PMC5940835; doi:10.1038/s41598-018-25500-x)
Supplement: Supplementary file 1 — Table S1 [file 41598_2018_25500_MOESM1_ESM.doc]

**Molecular targets of Chinese herbs: a clinical study of metastatic colorectal cancer based on network pharmacology**

Hongxu Zhu^1&^, Jian Hao^1&^, Yangyang Niu^2&^, Dan Liu^1^, Dan Chen^3^, Xiongzhi Wu^4^*

^&^These authors contributed equally to this study and share first authorship

1. Tianjin Medical University Cancer Institute and Hospital, National Clinical Research Center for Cancer, Key Laboratory of Cancer Prevention and Therapy, Tianjin, 300060, China

Hongxu [Zhu, 15172960361@163.com](mailto:Zhu,15172960361@163.com); Jian Hao, [haojian1111520@126.com](mailto:haojian1111520@126.com); Dan Liu, 1187740949@qq.com;

1. Tianjin Children’s Hospital, Tianjin, 300134, China

Yangyang Niu, [niuyangyang2013@163.com;](mailto:niuyangyang2013@163.com;)

3. Department of Pharmacology, School of Basic Medical Sciences, Tianjin Medical University, Tianjin, Qi-Xiang-Tai Road, Tianjin 300070, China

Dan Chen, ilvcd@163.com;

4. Zhong-Shan-Men Inpatient Department, Tianjin Medical University Cancer Institute and Hospital, Tianjin, 300060, China.

Xiongzhi Wu, wuxiongzhi@163.com.

***Correspondence to**: Xiong-Zhi Wu, Huan-Hu-Xi Road, Ti-Yuan-Bei, He-Xi District, Zhong-Shan-Men Inpatient Department, Tianjin Medical University Cancer Institute and Hospital, Tianjin, 300060, China. Telephone: +86-22-23921723 Fax: +86-22-23921723. E-mail address: wuxiongzhi@163.com.

**Table S1. Details of the baseline characteristics of patients with mCRC.**

| **Variable** | | **Non-CHM group N=144** | **CHM group N=78** | ***P* value** |
| --- | --- | --- | --- | --- |
|  |  |  |  |  |
| **Gender** Male/Female | | 84/60 | 52/26 | 0.224 |
|  |  |  |  |  |
| **Age(year)** | ≤40 | 6 | 8 | 0.104 |
|  | 40-60 | 73 | 43 |  |
|  | ≥60 | 65 | 27 |  |
| **Smoking**  Yes/no | | 50/94 | 20/58 | 0.164 |
|  |  |  |  |  |
| **Family history**  Yes/No | | 22/122 | 12/66 | 0.983 |
|  |  |  |  |  |
| **Tumor site**  Colon/Rectum | | 75/69 | 32/46 | 0.115 |
|  |  |  |  |  |
| **Primary tumor size**  ≤40/>4cm | | 49/95 | 27/51 | 0.730 |
|  |  |  |  |  |
| **Differentiated degree** | High | 6 | 4 | 0.276 |
|  | Middle | 107 | 64 |  |
|  | Poor | 31 | 10 |  |
| **Invaded the serous membrane**  Yes/No | | 113/31 | 55/23 | 0.187 |
|  |  |  |  |  |
| **Pathological type(adenocarcin-oma)** | Tubular | 112 | 64 | 0.482 |
|  | Mucinous | 25 | 9 |  |
|  | Papillary | 7 | 5 |  |
| **CA19-9**  High/Normal | | 47/97 | 28/50 | 0.624 |
|  |  |  |  |  |
| **CA24-2**  High/Normal | | 73/71 | 32/46 | 0.168 |
|  |  |  |  |  |
| **CA72-4**  High/Normal | | 54/90 | 21/57 | 0.112 |
|  |  |  |  |  |
| **CEA**  High/Normal | | 78/66 | 51/27 | 0.106 |
|  |  |  |  |  |
| **Lymph node**  **metastases** | No | 69 | 39 | 0.430 |
|  | 1-3 | 49 | 30 |  |
|  | ≥4 | 26 | 9 |  |
| **Systemic chemotherapy**  Yes/No | | 122/22 | 72/6 | 0.104 |
|  |  |  |  |  |
| **Radiotherapy**  Yes/No | | 24/120 | 21/57 | 0.070 |
|  |  |  |  |  |
| **R0 after metastasis**  Yes/No | | 44/100 | 27/51 | 0.536 |
|  |  |  |  |  |

**CEA**, carcinoembryonic antigen; **CA-199**, carbohydrate antigen 199; **CA-242**, carbohydrate antigen 242; **CA-724**, carbohydrate antigen 724; **R0**, complete resection; **CHM**, Chinese herbal medicine
