# Supplementary material for: Molecular targets of Chinese herbs: a clinical study of metastatic colorectal cancer based on network pharmacology
Source: Sci Rep. 2018 May 8;8:7238. doi: 10.1038/s41598-018-25500-x (PMC5940835; doi:10.1038/s41598-018-25500-x)
Supplement: Supplementary file 2 — Table S2 [file 41598_2018_25500_MOESM2_ESM.doc]

**Molecular targets of Chinese herbs: a clinical study of metastatic colorectal cancer based on network pharmacology**

Hongxu Zhu^1&^, Jian Hao^1&^, Yangyang Niu^2&^, Dan Liu^1^, Dan Chen^3^, Xiongzhi Wu^4^*

^&^These authors contributed equally to this study and share first authorship

1. Tianjin Medical University Cancer Institute and Hospital, National Clinical Research Center for Cancer, Key Laboratory of Cancer Prevention and Therapy, Tianjin, 300060, China

Hongxu [Zhu, 15172960361@163.com](mailto:Zhu,15172960361@163.com); Jian Hao, [haojian1111520@126.com](mailto:haojian1111520@126.com); Dan Liu, 1187740949@qq.com;

1. Tianjin Children’s Hospital, Tianjin, 300134, China

Yangyang Niu, [niuyangyang2013@163.com;](mailto:niuyangyang2013@163.com;)

3. Department of Pharmacology, School of Basic Medical Sciences, Tianjin Medical University, Tianjin, Qi-Xiang-Tai Road, Tianjin 300070, China

Dan Chen, ilvcd@163.com;

4. Zhong-Shan-Men Inpatient Department, Tianjin Medical University Cancer Institute and Hospital, Tianjin, 300060, China.

Xiongzhi Wu, wuxiongzhi@163.com.

***Correspondence to**: Xiong-Zhi Wu, Huan-Hu-Xi Road, Ti-Yuan-Bei, He-Xi District, Zhong-Shan-Men Inpatient Department, Tianjin Medical University Cancer Institute and Hospital, Tianjin, 300060, China. Telephone: +86-22-23921723 Fax: +86-22-23921723. E-mail address: wuxiongzhi@163.com.

**Table S2.** **Potential Ingredients Predicted in the 18 Herbs and Their Candidate Targets for CRC Treatment.**

| **Herbs** | **MOL** | **Molecular name** | **Target name** | **SYMBOL** |
| --- | --- | --- | --- | --- |
| ***Herba Patriniae*** | HP1 | apigenin | RAC-alpha serine/threonine-protein kinase | AKT1 |
| ***败酱草*** | HP2 | bolusanthol B | Peroxisome proliferator activated receptor gamma | PPARG |
|  | HP3 | kaempferol | RAC-alpha serine/threonine-protein kinase | AKT1 |
|  | HP3 | kaempferol | Peroxisome proliferator activated receptor gamma | PPARG |
|  | HP4 | luteolin | RAC-alpha serine/threonine-protein kinase | AKT1 |
|  | HP4 | luteolin | Epidermal growth factor receptor | EGFR |
|  | HP4 | luteolin | Receptor tyrosine-protein kinase erbB-2 | ERBB2 |
|  | HP4 | luteolin | Peroxisome proliferator-activated receptor gamma | PPARG |
|  | HP5 | quercetin | RAC-alpha serine/threonine-protein kinase | AKT1 |
|  | HP5 | quercetin | Epidermal growth factor receptor | EGFR |
|  | HP5 | quercetin | Receptor tyrosine-protein kinase erbB-2 | ERBB2 |
|  | HP5 | quercetin | Receptor tyrosine-protein kinase erbB-3 | ERBB3 |
|  | HP5 | quercetin | Peroxisome proliferator activated receptor gamma | PPARG |
|  | HP6 | ursolic acid | Protein kinase C gamma type | PRKCG |
| ***Schisandrae Chinensis Fructus*** | SCF1 | protocatechuic acid | Protein kinase C gamma type | PRKCG |
| ***五味子*** | SCF2 | Arnebin 7 | Peroxisome proliferator activated receptor gamma | PPARG |
|  | SCF3 | Gomisin T | Peroxisome proliferator activated receptor gamma | PPARG |
|  | SCF4 | Schisanhenol | Peroxisome proliferator activated receptor gamma | PPARG |
| ***Xanthii Fructus*** | XF1 | (-)-nopinene | Retinoic acid receptor RXR-alpha | RXRA |
| ***苍耳子*** | XF2 | (+)-Syringaresinol | DNA topoisomerase II | TOP2 |
|  | XF3 | (2R,3R)-3-(4-hydroxy-3-methoxy-phenyl)-5-methoxy-2-methylol-2,3-dihydropyrano[5,6-h][1,4]benzodioxin-9-one | DNA topoisomerase II | TOP2 |
|  | XF3 | (2R,3R)-3-(4-hydroxy-3-methoxy-phenyl)-5-methoxy-2-methylol-2,3-dihydropyrano[5,6-h][1,4]benzodioxin-9-one | Vascular endothelial growth factor receptor 2 | VEGFR2 |
|  | XF4 | (3R,4aR,8aR)-3-isopropenyl-5,8a-dimethyl-2,3,4,4a,7,8-hexahydro-1H-naphthalene | Retinoic acid receptor RXR-alpha | RXRA |
|  | XF5 | (L)-alpha-Terpineol | DNA topoisomerase II | TOP2 |
|  | XF6 | (R)-linalool | DNA topoisomerase II | TOP2 |
|  | XF6 | (R)-linalool | Vascular endothelial growth factor receptor 2 | VEGFR2 |
|  | XF7 | (R)-p-Menth-1-en-4-ol | Retinoic acid receptor RXR-alpha | RXRA |
|  | XF8 | aloe-emodin | Tumor necrosis factor | TNF |
|  | XF9 | beta-caryophyllene | Retinoic acid receptor RXR-alpha | RXRA |
|  | XF10 | beta-Selinene | Retinoic acid receptor RXR-alpha | RXRA |
|  | XF11 | CADINENE | Retinoic acid receptor RXR-alpha | RXRA |
|  | XF12 | caffeic acid | Tumor necrosis factor | TNF |
|  | XF13 | EIC | Retinoic acid receptor RXR-alpha | RXRA |
|  | XF14 | emodin | Peroxisome proliferator-activated receptor gamma | PPARG |
|  | XF14 | emodin | Tumor necrosis factor | TNF |
|  | XF14 | emodin | DNA topoisomerase II | TOP2 |
|  | XF14 | emodin | Vascular endothelial growth factor receptor 1 | VEGFR1 |
|  | XF14 | emodin | Vascular endothelial growth factor receptor 2 | VEGFR2 |
|  | XF14 | emodin | Vascular endothelial growth factor receptor 3 | VEGFR3 |
|  | XF15 | FUM | Proto-oncogene tyrosine-protein kinase SRC | SRC |
|  | XF16 | hydroquinone | Tumor necrosis factor | TNF |
|  | XF17 | L-Bornyl acetate | DNA topoisomerase II | TOP2 |
|  | XF18 | Ligla | Retinoic acid receptor RXR-alpha | RXRA |
|  | XF19 | Linoleic | Retinoic acid receptor RXR-alpha | RXRA |
|  | XF20 | linolenic acid | Retinoic acid receptor RXR-alpha | RXRA |
|  | XF21 | methyl palmitate | Tumor necrosis factor | TNF |
|  | XF22 | MLT | Proto-oncogene tyrosine-protein kinase SRC | SRC |
|  | XF23 | muurolene | Retinoic acid receptor RXR-alpha | RXRA |
|  | XF24 | oleic acid | Peroxisome proliferator-activated receptor gamma | PPARG |
|  | XF24 | oleic acid | Retinoic acid receptor RXR-alpha | RXRA |
|  | XF24 | oleic acid | Superoxide dismutase [Cu-Zn] | SOD1 |
|  | XF25 | Ononin | Peroxisome proliferator activated receptor gamma | PPARG |
|  | XF25 | Ononin | Vascular endothelial growth factor receptor 2 | VEGFR2 |
|  | XF26 | palmitic acid | Tumor necrosis factor | TNF |
|  | XF27 | phytol | Retinoic acid receptor RXR-alpha | RXRA |
|  | XF28 | protocatechuic acid | Protein kinase C gamma type | PRKCG |
|  | XF29 | Sitogluside | Retinoic acid receptor RXR-alpha | RXRA |
|  | XF30 | stearic acid | Retinoic acid receptor RXR-alpha | RXRA |
|  | XF31 | Stigmasterol | Retinoic acid receptor RXR-alpha | RXRA |
|  | XF32 | succinic acid | Proto-oncogene tyrosine-protein kinase SRC | SRC |
| ***Radix Paeoniae Rubra*** | RPR1 | (+)-catechin | Retinoic acid receptor RXR-alpha | RXRA |
| ***赤芍*** | RPR2 | 3,4,5-trihydroxybenzoic acid | DNA topoisomerase II | TOP2 |
|  | RPR3 | baicalein | RAC-alpha serine/threonine-protein kinase | AKT1 |
|  | RPR4 | BOX | Retinoic acid receptor RXR-alpha | RXRA |
|  | RPR5 | butylated hydroxytoluene | Retinoic acid receptor RXR-alpha | RXRA |
|  | RPR5 | butylated hydroxytoluene | Vascular endothelial growth factor receptor 2 | VEGFR2 |
|  | RPR6 | EIC | Retinoic acid receptor RXR-alpha | RXRA |
|  | RPR7 | eugenol | Serine/threonine-protein phosphatase 2B catalytic subunit alpha isoform | AKT1 |
|  | RPR8 | hexanal | Tumor necrosis factor | TNF |
|  | RPR9 | hydroquinone | Tumor necrosis factor | TNF |
|  | RPR10 | lauric acid | RAC-alpha serine/threonine-protein kinase | AKT1 |
|  | RPR11 | METHYL LINOLEATE | Retinoic acid receptor RXR-alpha | RXRA |
|  | RPR12 | methyl palmitate | Tumor necrosis factor | TNF |
|  | RPR13 | oleic acid | Receptor tyrosine-protein kinase erbB-2 | ERBB2 |
|  | RPR13 | oleic acid | Peroxisome proliferator-activated receptor gamma | PPARG |
|  | RPR13 | oleic acid | Retinoic acid receptor RXR-alpha | RXRA |
|  | RPR14 | paeoniflorin | Tumor necrosis factor | TNF |
|  | RPR15 | paeonol | RAC-alpha serine/threonine-protein kinase | AKT1 |
|  | RPR15 | paeonol | Tumor necrosis factor | TNF |
|  | RPR16 | Paeonolide | DNA topoisomerase II | TOP2 |
|  | RPR17 | palmitic acid | Tumor necrosis factor | TNF |
|  | RPR18 | Sitogluside | Retinoic acid receptor RXR-alpha | RXRA |
|  | RPR19 | Stigmasterol | Retinoic acid receptor RXR-alpha | RXRA |
|  | RPR20 | sucrose | Peroxisome proliferator-activated receptor gamma | PPARG |
|  | RPR21 | Tetracosane | Retinoic acid receptor RXR-alpha | RXRA |
| ***Angelicae Sinensis Radix*** | ASR1 | ()-alpha-Terpineol | Retinoic acid receptor RXR-alpha | RXRA |
| ***当归*** | ASR2 | ()-Cuparene | Retinoic acid receptor RXR-alpha | RXRA |
|  | ASR3 | (1R,4R,5S)-4-isopropenyl-1,8-dimethylspiro[4.5]dec-8-ene | Retinoic acid receptor RXR-alpha | RXRA |
|  | ASR4 | (1S,4aR,8aR)-1-isopropyl-7-methyl-4-methylene-2,3,4a,5,6,8a-hexahydro-1H-naphthalene | Retinoic acid receptor RXR-alpha | RXRA |
|  | ASR5 | 7,10-PENTADECADIYNOIC ACID | Retinoic acid receptor RXR-alpha | RXRA |
|  | ASR6 | Acoradiene | Retinoic acid receptor RXR-alpha | RXRA |
|  | ASR7 | BdPh | Retinoic acid receptor RXR-alpha | RXRA |
|  | ASR8 | beta-Chamigrene | Retinoic acid receptor RXR-alpha | RXRA |
|  | ASR9 | beta-Selinene | Retinoic acid receptor RXR-alpha | RXRA |
|  | ASR10 | BUA | Proto-oncogene tyrosine-protein kinase SRC | SRC |
|  | ASR11 | CADINENE | Retinoic acid receptor RXR-alpha | RXRA |
|  | ASR12 | CHEBI:7 | DNA topoisomerase II | TOP2 |
|  | ASR13 | InChI=1/C15H24/c1-10-7-8-15-9-12(10)14(3,4)13(15)6-5-11(15)2/h7,11-13H,5-6,8-9H2,1-4H | Retinoic acid receptor RXR-alpha | RXRA |
|  | ASR14 | palmitic acid | Tumor necrosis factor | TNF |
|  | ASR15 | Sitogluside | Retinoic acid receptor RXR-alpha | RXRA |
|  | ASR16 | Stigmasterol | Retinoic acid receptor RXR-alpha | RXRA |
|  | ASR17 | succinic acid | Proto-oncogene tyrosine-protein kinase SRC | SRC |
|  | ASR18 | α-acoradiene | Retinoic acid receptor RXR-alpha | RXRA |
| ***Lycii Fructus*** | LF1 | 1,6-dimethyl-1-isopropyl-1,2,3,4,4a,7-hexahydronaphthalene | Retinoic acid receptor RXR-alpha | RXRA |
| ***枸杞子*** | LF2 | 4-[(Z,1R)-3-(4-methoxyphenyl)-1-vinylprop-2-enyl]phenol | Retinoic acid receptor RXR-alpha | RXRA |
|  | LF3 | 7-O-Methylluteolin-6-C-beta-glucoside_qt | DNA topoisomerase II | TOP2 |
|  | LF4 | citric acid | Proto-oncogene tyrosine-protein kinase SRC | SRC |
|  | LF5 | copaene | Retinoic acid receptor RXR-alpha | RXRA |
|  | LF6 | DBP | Retinoic acid receptor RXR-alpha | RXRA |
|  | LF7 | delta-amorphene | Retinoic acid receptor RXR-alpha | RXRA |
|  | LF8 | EIC | Retinoic acid receptor RXR-alpha | RXRA |
|  | LF9 | glycitein | Peroxisome proliferator activated receptor gamma | PPARG |
|  | LF9 | glycitein | Retinoic acid receptor RXR-alpha | RXRA |
|  | LF10 | lauric acid | RAC-alpha serine/threonine-protein kinase | AKT1 |
|  | LF11 | Linoleic | Retinoic acid receptor RXR-alpha | RXRA |
|  | LF12 | lupeol | Superoxide dismutase [Cu-Zn] | SOD1 |
|  | LF13 | Methyl linolelaidate | Retinoic acid receptor RXR-alpha | RXRA |
|  | LF14 | methyl palmitate | Tumor necrosis factor | TNF |
|  | LF15 | MLT | Proto-oncogene tyrosine-protein kinase SRC | SRC |
|  | LF16 | myristic acid | Rhinovirus coat protein | RXRA |
|  | LF17 | OXL | Proto-oncogene tyrosine-protein kinase SRC | SRC |
|  | LF18 | paeonol | RAC-alpha serine/threonine-protein kinase | AKT1 |
|  | LF18 | paeonol | Tumor necrosis factor | TNF |
|  | LF19 | palmitic acid | Tumor necrosis factor | TNF |
|  | LF20 | Physcion | Retinoic acid receptor RXR-alpha | RXRA |
|  | LF20 | Physcion | DNA topoisomerase II | TOP2 |
|  | LF21 | Physcion-8-O-beta-D-gentiobioside | DNA topoisomerase II | TOP2 |
|  | LF22 | quercetin | Vascular endothelial growth factor A | VEGFA |
|  | LF22 | quercetin | RAC-alpha serine/threonine-protein kinase | AKT1 |
|  | LF22 | quercetin | Receptor tyrosine-protein kinase erbB-2 | ERBB2 |
|  | LF22 | quercetin | Receptor tyrosine-protein kinase erbB-3 | ERBB3 |
|  | LF22 | quercetin | Peroxisome proliferator activated receptor gamma | PPARG |
|  | LF22 | quercetin | Retinoic acid receptor RXR-alpha | RXRA |
|  | LF22 | quercetin | Superoxide dismutase [Cu-Zn] | SOD1 |
|  | LF22 | quercetin | Tumor necrosis factor | TNF |
|  | LF22 | quercetin | DNA topoisomerase 1 | TOP1 |
|  | LF22 | quercetin | DNA topoisomerase II | TOP2 |
|  | LF23 | rutin | Superoxide dismutase [Cu-Zn] | SOD1 |
|  | LF23 | rutin | Tumor necrosis factor | TNF |
|  | LF23 | rutin | DNA topoisomerase II | TOP2 |
|  | LF24 | Stigmasterol | Retinoic acid receptor RXR-alpha | RXRA |
|  | LF25 | Tetracosane | Retinoic acid receptor RXR-alpha | RXRA |
|  | LF26 | tetradecane | Tumor necrosis factor | TNF |
| ***Eriocauli Flos*** | EF1 | 1,3,6-trihydroxy-2,5,7-trimethoxyxanthone | DNA topoisomerase II | TOP2 |
| ***谷精草*** | EF2 | 2-Benzo[1,3]dioxol-5-yl-5,7-dimethoxy-chroman | Retinoic acid receptor RXR-alpha | RXRA |
|  | EF3 | 7,30-dihydroxy-5,40,50-trimethoxyisoflavone | DNA topoisomerase II | TOP2 |
|  | EF4 | DBP | Retinoic acid receptor RXR-alpha | RXRA |
|  | EF5 | Linoleic | Retinoic acid receptor RXR-alpha | RXRA |
|  | EF6 | oleic acid | Retinoic acid receptor RXR-alpha | RXRA |
|  | EF7 | palmitic acid | Tumor necrosis factor | TNF |
|  | EF8 | Patuletin | DNA topoisomerase II | TOP2 |
|  | EF9 | quercetagetin | Peroxisome proliferator activated receptor gamma | PPARG |
|  | EF9 | quercetagetin | DNA topoisomerase II | TOP2 |
|  | EF10 | quercetin | RAC-alpha serine/threonine-protein kinase | AKT1 |
|  | EF10 | quercetin | Epidermal growth factor receptor | EGFR |
|  | EF10 | quercetin | Receptor tyrosine-protein kinase erbB-2 | ERBB2 |
|  | EF10 | quercetin | Receptor tyrosine-protein kinase erbB-3 | ERBB3 |
|  | EF10 | quercetin | Peroxisome proliferator-activated receptor gamma | PPARG |
|  | EF10 | quercetin | Retinoic acid receptor RXR-alpha | RXRA |
|  | EF10 | quercetin | Superoxide dismutase [Cu-Zn] | SOD1 |
|  | EF10 | quercetin | Tumor necrosis factor | TNF |
|  | EF10 | quercetin | DNA topoisomerase 1 | TOP1 |
|  | EF10 | quercetin | DNA topoisomerase II | TOP2 |
|  | EF10 | quercetin | Vascular endothelial growth factor A | VEGFA |
|  | EF11 | toralac-tone-9-o-b-d-glucopyranoside | DNA topoisomerase II | TOP2 |
| Magnolia Officinalis Rehd Et Wils． | (-)-alpha-cedrene | MO1 | Retinoic acid receptor RXR-alpha | RXRA |
| ***厚朴*** | (-)-nopinene | MO2 | Retinoic acid receptor RXR-alpha | RXRA |
|  | (L)-alpha-Terpineol | MO3 | DNA topoisomerase II | TOP2 |
|  | (R)-linalool | MO4 | DNA topoisomerase II | TOP2 |
|  | (R)-p-Menth-1-en-4-ol | MO5 | Retinoic acid receptor RXR-alpha | RXRA |
|  | (Z,Z)-farnesol | MO6 | Retinoic acid receptor RXR-alpha | RXRA |
|  | alpha-humulene | MO7 | Tumor necrosis factor | TNF |
|  | BB_NC-0668 | MO8 | Retinoic acid receptor RXR-alpha | RXRA |
|  | beta-Chamigrene | MO9 | Retinoic acid receptor RXR-alpha | RXRA |
|  | beta-Selinene | MO10 | Retinoic acid receptor RXR-alpha | RXRA |
|  | BU3 | MO11 | Proto-oncogene tyrosine-protein kinase SRC | SRC |
|  | CHEBI:7 | MO12 | DNA topoisomerase II | TOP2 |
|  | -cis-.beta.-Elemene diastereomer | MO13 | Retinoic acid receptor RXR-alpha | RXRA |
|  | DBP | MO14 | Retinoic acid receptor RXR-alpha | RXRA |
|  | D-Camphene | MO15 | DNA topoisomerase II | TOP2 |
|  | EIC | MO16 | Retinoic acid receptor RXR-alpha | RXRA |
|  | Eucalyptol | MO17 | Retinoic acid receptor RXR-alpha | RXRA |
|  | honokiol | MO18 | Peroxisome proliferator activated receptor gamma | PPARG |
|  | honokiol | MO18 | Retinoic acid receptor RXR-alpha | RXRA |
|  | L-Bornyl acetate | MO19 | DNA topoisomerase II | TOP2 |
|  | Magnolol | MO20 | Peroxisome proliferator activated receptor gamma | PPARG |
|  | Magnolol | MO20 | Retinoic acid receptor RXR-alpha | RXRA |
|  | METHYL LINOLEATE | MO21 | Retinoic acid receptor RXR-alpha | RXRA |
|  | Methyl linolelaidate | MO22 | Retinoic acid receptor RXR-alpha | RXRA |
|  | methyl palmitate | MO23 | Tumor necrosis factor | TNF |
|  | muurolene | MO24 | Retinoic acid receptor RXR-alpha | RXRA |
|  | paeonol | MO25 | RAC-alpha serine/threonine-protein kinase | AKT1 |
|  | paeonol | MO25 | Tumor necrosis factor | TNF |
|  | palmitic acid | MO26 | Tumor necrosis factor | TNF |
|  | phytol | MO27 | Retinoic acid receptor RXR-alpha | RXRA |
|  | Terpilene | MO28 | Retinoic acid receptor RXR-alpha | RXRA |
|  | Tetracosane | MO29 | Retinoic acid receptor RXR-alpha | RXRA |
|  | tetradecane | MO30 | Tumor necrosis factor | TNF |
| ***Cassiae Semen*** | CaS1 | 1,6,7-Trihydroxy-3-methoxy-anthraquinone | DNA topoisomerase II | TOP2 |
| ***决明子*** | CaS2 | aloe-emodin | Protein kinase C alpha type | AKT1 |
|  | CaS2 | aloe-emodin | Tumor necrosis factor | TNF |
|  | CaS3 | Anthraglycoside A | DNA topoisomerase II | TOP2 |
|  | CaS4 | aurantio-obtusin,6-o-beta-d-glucoside_qt | DNA topoisomerase II | TOP2 |
|  | CaS4 | aurantio-obtusin,6-o-beta-d-glucoside_qt | Peroxisome proliferator activated receptor gamma | PPARG |
|  | CaS5 | BOX | Retinoic acid receptor RXR-alpha | RXRA |
|  | CaS6 | Cassiaside A | DNA topoisomerase II | TOP2 |
|  | CaS7 | EIC | Retinoic acid receptor RXR-alpha | RXRA |
|  | CaS8 | emodin | DNA topoisomerase II | TOP2 |
|  | CaS8 | emodin | Peroxisome proliferator-activated receptor gamma | PPARG |
|  | CaS8 | emodin | Protein kinase C delta type | AKT1 |
|  | CaS8 | emodin | Tumor necrosis factor | TNF |
|  | CaS8 | emodin | Vascular endothelial growth factor receptor 1 | VEGFR1 |
|  | CaS8 | emodin | Vascular endothelial growth factor receptor 2 | VEGFR2 |
|  | CaS8 | emodin | Vascular endothelial growth factor receptor 3 | VEGFR3 |
|  | CaS9 | Exceparl M-OL | Retinoic acid receptor RXR-alpha | RXRA |
|  | CaS10 | methyl palmitate | Tumor necrosis factor | TNF |
|  | CaS11 | Obtusifolin 2-glucoside | DNA topoisomerase II | TOP2 |
|  | CaS12 | Obtusin | DNA topoisomerase II | TOP2 |
|  | CaS13 | paeonol | RAC-alpha serine/threonine-protein kinase | AKT1 |
|  | CaS13 | paeonol | Tumor necrosis factor | TNF |
|  | CaS14 | Physcion | DNA topoisomerase II | TOP2 |
|  | CaS14 | Physcion | Retinoic acid receptor RXR-alpha | RXRA |
|  | CaS15 | Questin | DNA topoisomerase II | TOP2 |
|  | CaS15 | Questin | Retinoic acid receptor RXR-alpha | RXRA |
|  | CaS16 | Rubrofusarin-6-beta-gentiobioside | DNA topoisomerase II | TOP2 |
|  | CaS17 | Stigmasterol | Retinoic acid receptor RXR-alpha | RXRA |
|  | CaS18 | Torachrysone | Retinoic acid receptor RXR-alpha | RXRA |
| ***Portulacae Herba*** | PH1 | 5,7-dihydroxy-2-(3-hydroxy-4-methoxyphenyl)chroman-4-one | DNA topoisomerase II | TOP2 |
| ***马齿苋*** | PH2 | acetic acid | Beta-catenin | CTNNB1 |
|  | PH2 | acetic acid | Nitrogen regulation protein NR(I) | SIRT1 |
|  | PH2 | acetic acid | Proto-oncogene tyrosine-protein kinase SRC | SRC |
|  | PH2 | acetic acid | Ribonucleoside-diphosphate reductase large subunit | RRM1 |
|  | PH2 | acetic acid | Tumor necrosis factor | TNF |
|  | PH2 | acetic acid | Superoxide dismutase [Cu-Zn] | SOD1 |
|  | PH3 | apigenin | DNA topoisomerase II | TOP2 |
|  | PH3 | apigenin | Insulin-like growth factor 1 receptor | IGF1R |
|  | PH3 | apigenin | RAC-alpha serine/threonine-protein kinase | AKT1 |
|  | PH3 | apigenin | Tumor necrosis factor | TNF |
|  | PH3 | apigenin | Vascular endothelial growth factor A | VEGFA |
|  | PH4 | arachidonic acid | Peroxisome proliferator-activated receptor gamma | PPARG |
|  | PH4 | arachidonic acid | Platelet endothelial cell adhesion molecule | PECAM1 |
|  | PH4 | arachidonic acid | Retinoic acid receptor RXR-alpha | RXRA |
|  | PH5 | beta-carotene | Catenin beta-1 | CTNNB1 |
|  | PH5 | beta-carotene | RAC-alpha serine/threonine-protein kinase | AKT1 |
|  | PH5 | beta-carotene | Vascular endothelial growth factor A | VEGFA |
|  | PH6 | caffeic acid | Tumor necrosis factor | TNF |
|  | PH7 | citric acid | Proto-oncogene tyrosine-protein kinase SRC | SRC |
|  | PH8 | EIC | Retinoic acid receptor RXR-alpha | RXRA |
|  | PH9 | FUM | Proto-oncogene tyrosine-protein kinase SRC | SRC |
|  | PH10 | isobetanidin | DNA topoisomerase II | TOP2 |
|  | PH11 | kaempferol | DNA topoisomerase II | TOP2 |
|  | PH11 | kaempferol | Peroxisome proliferator activated receptor gamma | PPARG |
|  | PH11 | kaempferol | Serine/threonine-protein phosphatase 2B catalytic subunit alpha isoform | AKT1 |
|  | PH11 | kaempferol | Tumor necrosis factor | TNF |
|  | PH12 | lauric acid | RAC-alpha serine/threonine-protein kinase | AKT1 |
|  | PH13 | linolenic acid | Retinoic acid receptor RXR-alpha | RXRA |
|  | PH14 | lupeol | Superoxide dismutase [Cu-Zn] | SOD1 |
|  | PH15 | luteolin | DNA topoisomerase 1 | TOP1 |
|  | PH15 | luteolin | DNA topoisomerase 2-alpha | TOP2A |
|  | PH15 | luteolin | Epidermal growth factor receptor | EGFR |
|  | PH15 | luteolin | Hepatocyte growth factor receptor | MET |
|  | PH15 | luteolin | Peroxisome proliferator-activated receptor gamma | PPARG |
|  | PH15 | luteolin | RAC-alpha serine/threonine-protein kinase | AKT1 |
|  | PH15 | luteolin | Receptor tyrosine-protein kinase erbB-2 | ERBB2 |
|  | PH15 | luteolin | Tumor necrosis factor | TNF |
|  | PH15 | luteolin | Vascular endothelial growth factor A | VEGFA |
|  | PH16 | MLT | Proto-oncogene tyrosine-protein kinase SRC | SRC |
|  | PH17 | myricetin | DNA topoisomerase 1 | TOP1 |
|  | PH17 | myricetin | DNA topoisomerase II | TOP2 |
|  | PH17 | myricetin | Peroxisome proliferator activated receptor gamma | PPARG |
|  | PH17 | myricetin | Sodium/potassium-transporting ATPase subunit alpha-1 | ATP1A1 |
|  | PH17 | myricetin | Tumor necrosis factor | TNF |
|  | PH18 | nicotinic acid | Peroxisome proliferator-activated receptor gamma coactivator 1-alpha | PPARGC1A |
|  | PH19 | oleic acid | Peroxisome proliferator-activated receptor gamma | PPARG |
|  | PH19 | oleic acid | Receptor tyrosine-protein kinase erbB-2 | ERBB2 |
|  | PH19 | oleic acid | Retinoic acid receptor RXR-alpha | RXRA |
|  | PH19 | oleic acid | Superoxide dismutase [Cu-Zn] | SOD1 |
|  | PH20 | OXL | Proto-oncogene tyrosine-protein kinase SRC | SRC |
|  | PH20 | OXL | Ribonucleoside-diphosphate reductase large subunit | RRM1 |
|  | PH21 | palmitic acid | Tumor necrosis factor | TNF |
|  | PH22 | quercetin | DNA topoisomerase 1 | TOP1 |
|  | PH22 | quercetin | DNA topoisomerase 2-alpha | TOP2A |
|  | PH22 | quercetin | DNA topoisomerase II | TOP2 |
|  | PH22 | quercetin | Epidermal growth factor receptor | EGFR |
|  | PH22 | quercetin | Peroxisome proliferator activated receptor gamma | PPARG |
|  | PH22 | quercetin | RAC-alpha serine/threonine-protein kinase | AKT1 |
|  | PH22 | quercetin | Receptor tyrosine-protein kinase erbB-2 | ERBB2 |
|  | PH22 | quercetin | Tumor necrosis factor | TNF |
|  | PH22 | quercetin | Vascular endothelial growth factor A | VEGFA |
|  | PH22 | quercetin | Retinoic acid receptor RXR-alpha | RXRA |
|  | PH22 | quercetin | Superoxide dismutase [Cu-Zn] | SOD1 |
|  | PH23 | stearic acid | Retinoic acid receptor RXR-alpha | RXRA |
|  | PH24 | succinic acid | Proto-oncogene tyrosine-protein kinase SRC | SRC |
| ***Ranunculi Ternati Radix*** | RTR1 | bilobetin | Tumor necrosis factor | TNF |
| ***猫抓草*** | RTR2 | citric acid | Proto-oncogene tyrosine-protein kinase SRC | SRC |
|  | RTR3 | DBP | Retinoic acid receptor RXR-alpha | RXRA |
|  | RTR4 | DIBP | Retinoic acid receptor RXR-alpha | RXRA |
|  | RTR5 | EIC | Retinoic acid receptor RXR-alpha | RXRA |
|  | RTR6 | Etilevodopa | Peroxisome proliferator activated receptor gamma | PPARG |
|  | RTR7 | linolenic acid | Retinoic acid receptor RXR-alpha | RXRA |
|  | RTR8 | oleic acid | Receptor tyrosine-protein kinase erbB-2 | ERBB2 |
|  | RTR8 | oleic acid | Peroxisome proliferator-activated receptor gamma | PPARG |
|  | RTR8 | oleic acid | Retinoic acid receptor RXR-alpha | RXRA |
|  | RTR9 | palmitic acid | Tumor necrosis factor | TNF |
|  | RTR10 | stearic acid | Retinoic acid receptor RXR-alpha | RXRA |
|  | RTR11 | Stigmasterol | Retinoic acid receptor RXR-alpha | RXRA |
|  | RTR12 | succinic acid | Proto-oncogene tyrosine-protein kinase SRC | SRC |
|  | RTR13 | Tetracosane | Retinoic acid receptor RXR-alpha | RXRA |
| ***Aucklandiae Radix*** | AR1 | (-)-alpha-cedrene | Retinoic acid receptor RXR-alpha | RXRA |
| ***木香*** | AR2 | (1R,4R)-4-isopropyl-1,6-dimethyltetralin | Retinoic acid receptor RXR-alpha | RXRA |
|  | AR3 | (1R,5R,7S)-4,7-dimethyl-7-(4-methylpent-3-enyl)bicyclo[3.1.1]hept-3-ene | Retinoic acid receptor RXR-alpha | RXRA |
|  | AR4 | (L)-alpha-Terpineol | DNA topoisomerase II | TOP2 |
|  | AR5 | (R)-linalool | DNA topoisomerase II | TOP2 |
|  | AR5 | (R)-linalool | Vascular endothelial growth factor receptor 2 | VEGFR2 |
|  | AR6 | (R)-p-Menth-1-en-4-ol | Retinoic acid receptor RXR-alpha | RXRA |
|  | AR7 | alpha-humulene | Tumor necrosis factor | TNF |
|  | AR8 | butylated hydroxytoluene | Vascular endothelial growth factor receptor 2 | VEGFR2 |
|  | AR8 | butylated hydroxytoluene | Retinoic acid receptor RXR-alpha | RXRA |
|  | AR9 | -cis-.beta.-Elemene diastereomer | Retinoic acid receptor RXR-alpha | RXRA |
|  | AR10 | D-Camphene | DNA topoisomerase II | TOP2 |
|  | AR11 | EIC | Retinoic acid receptor RXR-alpha | RXRA |
|  | AR12 | Eremophilene | Retinoic acid receptor RXR-alpha | RXRA |
|  | AR13 | hexanal | Tumor necrosis factor | TNF |
|  | AR14 | IFP | Proto-oncogene tyrosine-protein kinase SRC | SRC |
|  | AR15 | Stigmasterol | Retinoic acid receptor RXR-alpha | RXRA |
| ***Taraxacum mongolicum Hand*** | TM1 | 2-Hydroxybenzoic acid | Superoxide dismutase [Cu-Zn] | SOD1 |
| ***蒲公英*** | TM2 | benzenecarboxylic acid | Retinoic acid receptor RXR-alpha | RXRA |
|  | TM3 | copper | Retinoic acid receptor RXR-alpha | RXRA |
|  | TM4 | L-ALFA-LYSOPHOSPHATIDYLCHOLINE, LAUROYL | RAC-alpha serine/threonine-protein kinase | AKT1 |
|  | TM5 | linolenic acid | Retinoic acid receptor RXR-alpha | RXRA |
|  | TM6 | palmitic acid | Tumor necrosis factor | TNF |
|  | TM7 | quercetin | DNA topoisomerase 1 | TOP1 |
|  | TM7 | quercetin | DNA topoisomerase II | TOP2 |
|  | TM7 | quercetin | Epidermal growth factor receptor | EGFR |
|  | TM7 | quercetin | Insulin-like growth factor II | IGF2 |
|  | TM7 | quercetin | Peroxisome proliferator activated receptor gamma | PPARG |
|  | TM7 | quercetin | Protein kinase C beta type | PRKCA |
|  | TM7 | quercetin | RAC-alpha serine/threonine-protein kinase | AKT1 |
|  | TM7 | quercetin | Receptor tyrosine-protein kinase erbB-2 | ERBB2 |
|  | TM7 | quercetin | Receptor tyrosine-protein kinase erbB-3 | ERBB3 |
|  | TM7 | quercetin | Retinoic acid receptor RXR-alpha | RXRA |
|  | TM7 | quercetin | Superoxide dismutase [Cu-Zn] | SOD1 |
|  | TM7 | quercetin | Transforming growth factor beta-1 | TGFB1 |
|  | TM7 | quercetin | Tumor necrosis factor | TNF |
|  | TM7 | quercetin | Vascular endothelial growth factor A | VEGFA |
|  | TM8 | rutin | DNA topoisomerase II | TOP2 |
|  | TM8 | rutin | Protein kinase C beta type | PRKCA |
|  | TM8 | rutin | Superoxide dismutase [Cu-Zn] | SOD1 |
|  | TM8 | rutin | Tumor necrosis factor | TNF |
|  | TM9 | sodium | Retinoic acid receptor RXR-alpha | RXRA |
|  | TM10 | stearic acid | Retinoic acid receptor RXR-alpha | RXRA |
|  | TM11 | vitamin b2 | DNA topoisomerase II | TOP2 |
|  | TM12 | β-sitosterol | DNA topoisomerase II | TOP2 |
| ***Selaginella Doederleinii Hieron*** | SDH1 | ACon1_001856 | DNA topoisomerase II | TOP2 |
| ***石上柏*** | SDH2 | apigenin | DNA topoisomerase II | TOP2 |
|  | SDH2 | apigenin | Insulin-like growth factor 1 receptor | IGF1R |
|  | SDH2 | apigenin | RAC-alpha serine/threonine-protein kinase | AKT1 |
|  | SDH2 | apigenin | Tumor necrosis factor | TNF |
|  | SDH2 | apigenin | Vascular endothelial growth factor A | VEGFA |
|  | SDH3 | stearic acid | Retinoic acid receptor RXR-alpha | RARA |
|  | SDH4 | Yangambin | DNA topoisomerase II | TOP2 |
| ***Fallopia multiflora*** | FM1 | emodin | Vascular endothelial growth factor receptor 2 | VEGFR2 |
| ***首乌藤*** | FM1 | emodin | DNA topoisomerase II | TOP2 |
|  | FM1 | emodin | Vascular endothelial growth factor receptor 1 | VEGFR1 |
|  | FM1 | emodin | Tumor necrosis factor | TNF |
|  | FM1 | emodin | Peroxisome proliferator-activated receptor gamma | PPARG |
|  | FM1 | emodin | Vascular endothelial growth factor receptor 3 | VEGFR3 |
| ***Radix Clematidis*** | RC1 | 2,3-Dihydro-4-hydroxy-2-indole-3-acetonitrile | Peroxisome proliferator activated receptor gamma | PPARG |
| ***威灵仙*** | RC2 | BOX | Retinoic acid receptor RXR-alpha | RXRA |
|  | RC3 | DBP | Retinoic acid receptor RXR-alpha | RXRA |
|  | RC4 | METHYL LINOLEATE | Retinoic acid receptor RXR-alpha | RXRA |
|  | RC5 | methyl palmitate | Tumor necrosis factor | TNF |
|  | RC6 | nonane | Tumor necrosis factor | TNF |
|  | RC7 | palmitic acid | Tumor necrosis factor | TNF |
|  | RC8 | Stigmasterol | Retinoic acid receptor RXR-alpha | RXRA |
| ***Agrimonia Eupatoria*** | AE1 | (-)-alpha-cedrene | Retinoic acid receptor RXR-alpha | RARA |
| ***仙鹤草*** | AE2 | (-)-nopinene | Retinoic acid receptor RXR-alpha | RARA |
|  | AE3 | (+)-catechin | Retinoic acid receptor RXR-alpha | RARA |
|  | AE4 | (L)-alpha-Terpineol | DNA topoisomerase II | TOP2 |
|  | AE5 | (R)-linalool | DNA topoisomerase II | TOP2 |
|  | AE5 | (R)-linalool | Vascular endothelial growth factor receptor 2 | VEGFR2 |
|  | AE6 | 3,4,5-trihydroxybenzoic acid | DNA topoisomerase II | TOP2 |
|  | AE7 | 3R-hydroxy-butanoic acid | Proto-oncogene tyrosine-protein kinase SRC | SRC |
|  | AE8 | apigenin | DNA topoisomerase II | TOP2 |
|  | AE8 | apigenin | Insulin-like growth factor 1 receptor | IGF1R |
|  | AE8 | apigenin | RAC-alpha serine/threonine-protein kinase | **AKT1** |
|  | AE8 | apigenin | Sodium/potassium-transporting ATPase subunit gamma | ATP1A1 |
|  | AE8 | apigenin | Tumor necrosis factor | TNF |
|  | AE8 | apigenin | Vascular endothelial growth factor A | VEGFA |
|  | AE8 | apigenin | Interferon gamma | IFNG |
|  | AE9 | caffeic acid | Tumor necrosis factor | TNF |
|  | AE9 | caffeic acid | Insulin-like growth factor II | IGF2 |
|  | AE10 | D-Camphene | DNA topoisomerase II | TOP2 |
|  | AE11 | ellagic acid | Vascular endothelial growth factor A | VEGFA |
|  | AE11 | ellagic acid | Insulin-like growth factor II | IGF2 |
|  | AE12 | Hyperin | DNA topoisomerase II | TOP2 |
|  | AE13 | kaempferol | DNA topoisomerase II | TOP2 |
|  | AE13 | kaempferol | Peroxisome proliferator activated receptor gamma | PPARG |
|  | AE13 | kaempferol | Serine/threonine-protein phosphatase 2B catalytic subunit alpha isoform | AKT1 |
|  | AE13 | kaempferol | Tumor necrosis factor | TNF |
|  | AE14 | lauric acid | RAC-alpha serine/threonine-protein kinase | AKT1 |
|  | AE14 | lauric acid | Tumor necrosis factor receptor superfamily member 5 | TNFRSF5 |
|  | AE15 | L-Limonen | DNA topoisomerase II | TOP2 |
|  | AE16 | luteolin | DNA topoisomerase 1 | TOP1 |
|  | AE16 | luteolin | DNA topoisomerase 2-alpha | TOP2 |
|  | AE16 | luteolin | Epidermal growth factor receptor | EGFR |
|  | AE16 | luteolin | Hepatocyte growth factor receptor | HGFR |
|  | AE16 | luteolin | Peroxisome proliferator-activated receptor gamma | PPARG |
|  | AE16 | luteolin | RAC-alpha serine/threonine-protein kinase | **AKT1** |
|  | AE16 | luteolin | Receptor tyrosine-protein kinase erbB-2 | ERBB2 |
|  | AE16 | luteolin | Tumor necrosis factor | TNF |
|  | AE16 | luteolin | Vascular endothelial growth factor A | VEGFA |
|  | AE16 | luteolin | Interferon gamma | IFNG |
|  | AE17 | osthol | Retinoic acid receptor RXR-alpha | RARA |
|  | AE18 | quercetin | DNA topoisomerase 1 | TOP1 |
|  | AE18 | quercetin | DNA topoisomerase 2-alpha | TOP2 |
|  | AE18 | quercetin | Epidermal growth factor receptor | EGFR |
|  | AE18 | quercetin | Insulin-like growth factor-binding protein 3 | IGFBP3 |
|  | AE18 | quercetin | Peroxisome proliferator activated receptor gamma | PPARG |
|  | AE18 | quercetin | RAC-alpha serine/threonine-protein kinase | **AKT1** |
|  | AE18 | quercetin | Receptor tyrosine-protein kinase erbB-2 | ERBB2 |
|  | AE18 | quercetin | Retinoic acid receptor RXR-alpha | RARA |
|  | AE18 | quercetin | Superoxide dismutase [Cu-Zn] | SOD1 |
|  | AE18 | quercetin | Tissue factor | TNF |
|  | AE18 | quercetin | Vascular endothelial growth factor A | VEGFA |
|  | AE18 | quercetin | Insulin-like growth factor II | IGF2 |
|  | AE18 | quercetin | Transforming growth factor beta-1 | TGFB1 |
|  | AE19 | ursolic acid | Platelet endothelial cell adhesion molecule | PECAM1 |
|  | AE19 | ursolic acid | Protein kinase C gamma type | PRKCG |
|  | AE19 | ursolic acid | Tumor necrosis factor | TNF |
|  | AE19 | ursolic acid | Vascular endothelial growth factor A | VEGFA |
|  | AE19 | ursolic acid | Granulocyte-macrophage colony-stimulating factor | CSF2 |
| ***Coicis Semen*** | Cos1 | EIC | Retinoic acid receptor RXR-alpha | RXRA |
| ***薏苡仁*** | Cos2 | hexanal | Tumor necrosis factor | TNF |
|  | Cos3 | linolenic acid | Retinoic acid receptor RXR-alpha | RXRA |
|  | Cos4 | METHYL LINOLEATE | Retinoic acid receptor RXR-alpha | RXRA |
|  | Cos5 | Methyl oleate | Retinoic acid receptor RXR-alpha | RXRA |
|  | Cos6 | oleic acid | Peroxisome proliferator-activated receptor gamma | PPARG |
|  | Cos6 | oleic acid | Receptor tyrosine-protein kinase erbB-2 | ERBB2 |
|  | Cos6 | oleic acid | Superoxide dismutase [Cu-Zn] | SOD1 |
|  | Cos6 | oleic acid | Retinoic acid receptor RXR-alpha | RXRA |
|  | Cos7 | Omaine | DNA topoisomerase II | TOP2 |
|  | Cos8 | palmitic acid | Tumor necrosis factor | TNF |
|  | Cos9 | stearic acid | Retinoic acid receptor RXR-alpha | RXRA |
|  | Cos10 | Stigmasterol | Retinoic acid receptor RXR-alpha | RXRA |
